# Guanosine-Based Supramolecular Particles for Enhanced Drug and Gene Delivery in Cell Culture

**DOI:** 10.1021/acsabm.5c00201

**Published:** 2025-06-10

**Authors:** Luis M. Negrón, Edwin Vázquez-Rosa, Luxene Belfleur, Tanya L. Díaz, Bismark Madera-Soto, Irving E. Vega, José M. Rivera

**Affiliations:** † Department of Chemistry, 2310 University of Puerto Rico at Río Piedras, San Juan 00925, PR; ‡ Molecular Sciences Research Center, University of Puerto Rico, San Juan 00926, PR; § Department of Chemistry, University of Puerto Rico at Cayey, Cayey 00736, PR; ∥ Department of Biology, University of Puerto Rico at Río Piedras, San Juan 00931, PR; ⊥ Department of Translational Science and Molecular Medicine College of Human Medicine, Michigan State University, Grand Rapids, Michigan 49503, United States

**Keywords:** self-assembly, supramolecular particles, guanosine derivatives, cellular uptake, gene delivery, drug delivery

## Abstract

Supramolecular hacky sacks (SHS) are a distinct class of self-assembled colloidal particles derived from guanosine (G) derivatives, engineered to support a wide range of cellular and therapeutic functions. In this study, we examine how variations in G-derivative composition influence SHS cellular uptake, intracellular trafficking, and functional efficacy. Confocal microscopy and flow cytometry reveal that uptake is highly dependent on particle composition, indicating selective engagement with specific cellular mechanisms. We show that SHS particles are biocompatible carriers capable of delivering both small molecules and genetic material: they successfully encapsulate and release doxorubicin with enhanced cytotoxic effects, and enable plasmid transfection with sustained expression of fluorescent proteins. These findings position SHS particles as a highly adaptable and effective supramolecular platform for drug and gene delivery. Their intrinsic biodegradability, ease of preparation, and tunable bioactivity highlight their strong potential for advancing biomedical applications.

## Graphical abstract

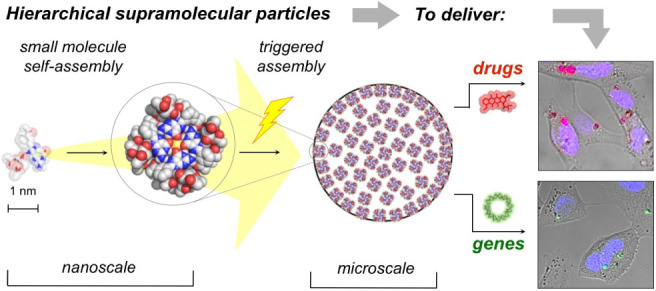

## 1 Introduction

In vitro cell culture studies using colloidal particle probes provide critical insights into cell biology, offering a foundational platform to evaluate systems designed for biomedical applications such as drug delivery.^1^ Colloidal multifunctional particles are increasingly recognized for their transformative potential in biology and medicine, serving as cellular probes^2–5^ and vehicles for delivering therapeutic agents and biologically active compounds.^6–10^ These systems enable precise modulation of cargo activity and deliver a diverse range of biologically active molecules, including small-molecule drugs,^11^ DNA and RNA therapeutics,^12–14^ proteins,^15,16^ and vaccine components.^17–19^ By combining precision and adaptability, colloidal particle-based probes offer unique advantages for advancing our understanding of cellular processes and driving innovation in biomedical applications.

Among the various particle delivery systems under development, lipid- and polymer-based systems are the most advanced and clinically promising. Lipid delivery systems have demonstrated clinical success,^20^ valued for their biocompatibility, optimization through structure–activity relationship (SAR) studies,^21^ and potential for oral administration.^22^ Polymer delivery systems, meanwhile, are rapidly approaching clinical applications,^23^ offering extensive customization options, including control over size, charge, and surface properties.^24^ However, despite their potential, lipid- and polymer-based particles face challenges such as complex preparative and formulation methods, which can hinder scalability and reproducibility.

Supramolecular chemistry provides powerful tools to help overcome these challenges by offering innovative strategies for designing more efficient and versatile delivery systems.^25,26^ Examples relevant to our study include the creation of organic nanoparticles with tunable cellular uptake efficiency based on adjustable size and surface traits such as lipophilicity,^27^ cyclodextrin-based supramolecular systems for efficient peptide delivery,^28^ and the assembly of FDA-approved anticancer drugs into stable nanostructures.^29^ These advancements illustrate the growing impact of supramolecular chemistry in biomedicine.

Inspired by such examples, our research focuses on developing assemblies of guanosine (G) derivatives, particularly colloidal particles termed supramolecular hacky sacks (SHS).^30–33^ SHS particles can form complexes with a wide range of biomedically relevant molecules, including drugs,^33^ DNA,^34^ and proteins.^32,35^ These particles are generated through the hierarchical self-assembly of G-derivatives into nanoscopic structures known as supramolecular G-quadruplexes (SGQs), which exhibit thermoresponsive behavior. Specifically, SHS particles form upon reaching a threshold temperature, undergoing a lower critical solution temperature (LCST) phase transition.^30^ As demonstrated later in this study, other stimuli, such as changes in pH, can also modulate SHS properties, including cargo release behavior. This unique mechanism yields colloidal structures with properties that are highly tunable through the molecular design of the G-derivatives, which can be synthesized with precision. This level of control facilitates structure–property relationship studies, enabling rational optimization of particle behavior, an advantage that is more difficult to achieve with other supramolecular, lipid- or polymer-based systems. These features distinguish SHS particles from existing supramolecular carriers and underscore their potential as a modular platform for biological and therapeutic applications.

Building on our prior work demonstrating the use of SHS particles for vaccine delivery,^35^ including delivery in mice studies,^36^ we sought to establish an initial profile through in vitro cell studies. This study explores the potential of SHS particles as a platform for developing multifunctional probes for fundamental biological research (Figure 1), while also laying the groundwork for their application as therapeutic delivery systems. This study represents an initial exploration of SHS particle properties in biological settings,^1^ setting the stage for future research to fully realize their potential as biological probes and in broader biomedical applications.

**Figure 1:**
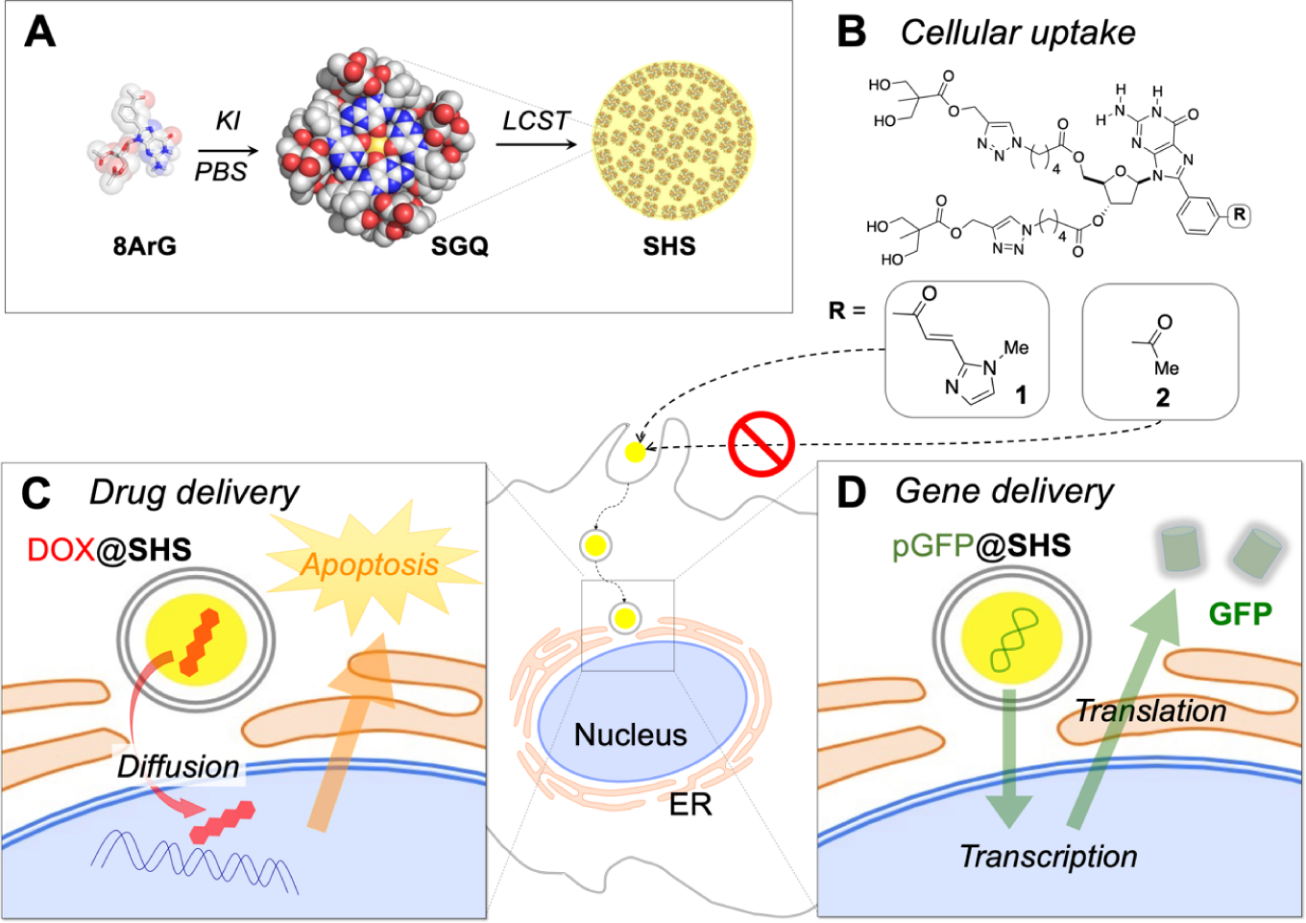
Overview of the study reported in this article. (A) Schematic representation of the hierarchical self-assembly of G-derivatives into SGQs and subsequent thermally induced self-assembly into SHS particles. The three biological properties evaluated here are (B) cellular uptake; (C) drug-delivery; and (D) gene-delivery. LCST means lower critical solution temperature and ER means endoplasmic reticulum.

## 2 Experimental Section

### 2.1 Chemical Compounds and Reagents

Compounds **1** and **2** were synthesized and characterized following the protocols previously reported by us.^30,31,37,38^ Doxorubicin (**DOX**) and amiloride were purchased from Sigma-Aldrich, Inc. Both Dextran Texas Red 3 kDa (**DTR-3**) and LysoTracker Deep Red were manufactured by Life Technologies, Inc. and used as received. The manufacturer’s reported absorption and fluorescence emission maxima for LysoTracker Deep Red are 647/668 nm, respectively (determined in aqueous buffer or methanol). The nuclear stains used were: (a) DAPI VECTASHIELD mounting medium (VECTOR Laboratories) for fixed cells; and (b) Hoechst 33342 (“NucBlue Live Ready Probes Reagent” kit from Life Technologies) for live cell imaging. All other reagents were also from commercial sources, and they were used without further purification or treatment.

### 2.2 Confocal Laser Scanning Microscopy (CLSM)

Confocal microscopy images were acquired using a Zeiss LSM 510 META system mounted on an Axiovision Z1 microscope at the Confocal Microscopy Facility of the University of Puerto Rico (CIF-UPR). Samples were excited at 561 nm using beam splitters configured as follows: MBS set to HFT 488/561, DBS1 as a mirror, and DBS2 set to NFT 565. Emission was detected in the 565–615 nm range. A 40×/0.75 EC Plan Neofluar objective was used for most samples, while a 63×/1.40 oil immersion Plan-Apochromat DIC M27 objective was used specifically for **DTR-3@SHS1**. For **SHS1** and **DTR-3@SHS1**, a band-pass (BP) 575–615 nm IR emission filter was applied, whereas a long-pass (LP) 575 nm emission filter was used for **DOX@SHS1**. Image analysis, including quantification of Mean Fluorescence Intensity (MFI), was conducted using NIS Elements software (version 6.10).

### 2.3 Flow Cytometry (FC)

Samples were analyzed in a BD Accuri C6 Cytometer with an excitation laser of 561 nm with an FL-2 (red) filter and all the measurements were performed in triplicate. Sample concentration was about 1 × 10^6^ per mL with a count rate of 10,000 events per second. All FC data were analyzed with the FCS Express software (version 4) except the median FL2-A statistics, which were performed with BD Accuri C6 Analysis software.

### 2.4 Zeta Potential (ZP)

After confirming the formation of SHS particles and their respective complexes (e.g., **SHS1**, **pCri@SHS1**) using microscopy techniques such as confocal laser scanning microscopy (CLSM) or scanning electron microscopy (SEM), zeta potential (ZP) measurements were carried out. ZP analysis was performed using a Zetasizer Nano ZS (model ZEN3600, Malvern Instruments Ltd.) operating in zeta potential mode at 25.0 ± 0.1 °C (Table S1). Phosphate-buffered saline (PBS, 1X, pH 7.4) was used as the dispersant, with a refractive index (RI) of 1.332 and a viscosity of 0.9074 cP at 30.0 °C. Measurements were processed using the Malvern software in automatic analysis mode, applying the Smoluchowski model with an F (κa) value of 1.50 at 25.0 °C.

### 2.5 Cells and Cell Culture

The human neuroblastoma (SH-SY5Y) and human embryonic kidney (HEK-293) cell lines were purchased from American Type Culture Collection (ATTC). Both cell lines were cultured in Dulbecco’s Eagle’s Medium/Nutrient Mixture F-12 (DMEM F-12), that was supplemented with 10% fetal bovine serum (FBS) (from Aldrich), 100 units/mL of penicillin, 100 μg/mL of streptomycin and 0.25 μg/mL of amphotericin B. Cells were incubated in humidified air containing 5.0% CO_2_ at 38.0 °C. Cells were seeded at 1 × 10^6^ cells/well in a 48-well plate (1.0 mL). For the movies, experiments with Lysotracker and transfection agents (SHS and lipofectamine) were performed in two-well-cubed coverslip chambers while for the rest of the images we used 48-well plates.

## 3 Results and Discussion

### 3.1 Preparation of the SHS Particles from G-Derivatives via SGQs

The synthesis of 8ArG derivatives **1** and **2** was performed as previously described by our group (Figure 2A).^30,31,38–40^ In high ionic strength solutions (0.7–4 M KI) at pH 7, either compound self-assembles into supramolecular G-quadruplexes (SGQs), which in turn give rise to colloidal SHS particles under mild thermal or pH triggers (Figure 2B).^34^ Specifically, SHS formation can be initiated either by raising the temperature to room temperature at pH 7 or by adjusting the pH from 5 to 7 at 25 °C. These SGQ-based particles are reversibly assembled; however, their morphology can be kinetically stabilized, or “fixed”, by lowering the ionic strength of the suspension after formation. This step effectively arrests particle dynamics and preserves colloidal structure under subsequent handling. The resulting SHS particles serve as versatile scaffolds whose encapsulation capabilities are discussed in detail in the following section (Figure 2C).^41^

**Figure 2:**
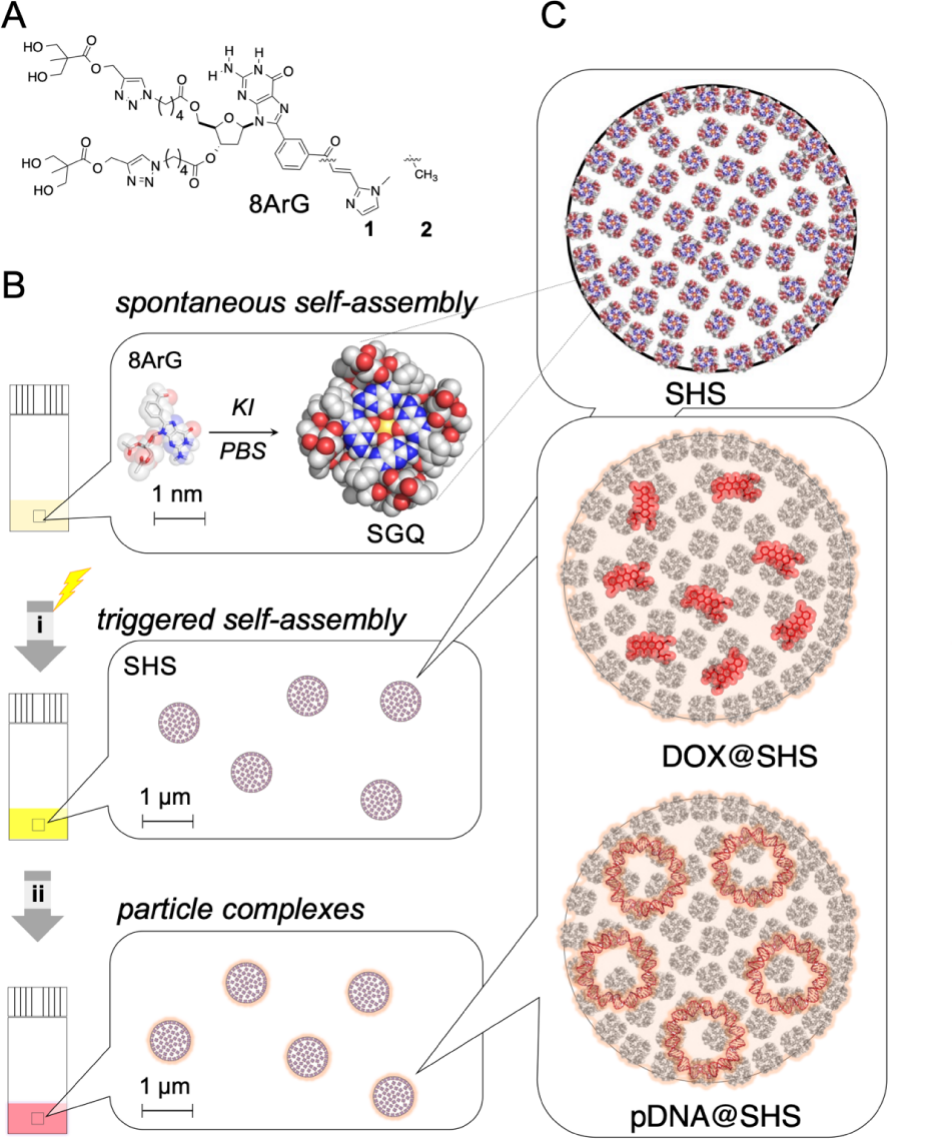
Architectural features of the SHS particles and schematic overview of the preparative protocol. (A) Chemical structures of G-derivatives (**1**, **2**); (B) general protocol for the preparation of the corresponding kinetic stabilized (“fixed”) particles and a (C) magnified version of part B.

### 3.2 Encapsulation of Biologically Relevant Cargo

We have recently reported multiple strategies for encapsulating biologically important molecules, such as drugs, proteins, and DNA, within SHS particles.^32–34^ To achieve this, we developed three encapsulation protocols (Figure 2). The first approach, an in situ method (Method 1), triggers SHS formation in the presence of the desired guest molecule.^40^ The other two approaches are variations of the osmotic gradient technique: in Method 2, the guest is incubated with the SHS colloid just prior to kinetic stabilization (fixing), while in Method 3, encapsulation occurs after the particles have been fixed. Once fixed, SHS particles become resistant to environmental perturbations (e.g., dilution, temperature changes), enabling their reliable use in biological systems.

These three methods provide flexibility to accommodate a wide range of guest molecules, with the choice depending on the properties and stability of the molecule of interest. Small molecules that tolerate high ionic strengths and elevated temperatures, such as **DOX**, can be encapsulated using any of the three methods. In practice, we typically favor Method 2 for **DOX** encapsulation because the fixing step can be coupled with a washing process to remove unencapsulated drug, resulting in purified and stable **DOX@SHS1** complexes. Method 3, although it generally results in lower encapsulation efficiency due to limited diffusional access, offers the unique advantage of overloading, wherein high guest concentrations in the incubation buffer drive increased encapsulation, an approach that is incompatible with Methods 1 and 2 due to the risk of disrupting SHS formation. In contrast, Method 3 is especially well suited for sensitive cargos, such as proteins,^32^ which may denature under the conditions required for particle formation in the first two methods.

### 3.3 Cellular Uptake of SHS1 and SHS2: FC and CLSM Studies

The biocompatibility of SHS particles is strongly supported by in vivo studies in mice, where SHS-formulated DNA vaccines produced no observable adverse effects throughout the immunization protocol while eliciting robust humoral and cellular immune responses.^36^ Building on this foundation, flow cytometry measurements in the present study confirm efficient cellular uptake of SHS particles and suggest potential selectivity among different cell types (Figure 2A–C). While **SHS1** exhibits intrinsic fluorescence due to the extended conjugation provided by the chalconyl moiety in derivative **1**, **SHS2** lacks this property (Figures S1–S3). To enable fluorescence-based tracking under consistent optical settings, we encapsulated Texas Red-labeled dextran (DTR-3) in both **SHS1** and **SHS2**. This labeling approach yields highly fluorescent complexes (**DTR-3@SHS1** and **DTR-3@SHS2**) that facilitate direct comparison of uptake dynamics using the same excitation/emission filters. Importantly, encapsulation of **DTR-3** does not involve covalent modification and has been shown to increase the mean fluorescence intensity (MFI) of SHS particles by several orders of magnitude.^33^ This strategy not only enhances visualization but also avoids introducing variables that could confound comparisons between **SHS1** and **SHS2**. Moreover, the use of **DTR-3**, a biocompatible dye commonly employed in biological assays, has been validated by us in prior studies as a reliable noncovalent label for SHS particles.^34^

Live-cell imaging reveals that the SHS particles are actively taken up by cells through membrane protrusions, indicative of an actin-driven process, followed by vesicular trafficking to the perinuclear region (Figures S4–S12 Movies S1 and S2). Some particles colocalize with lysosomal compartments, as suggested by Lysotracker Deep Red experiments (Figure S13).^42^ Based on these observations, we hypothesized that macropinocytosis is the primary uptake mechanism. This hypothesis is supported by experiments using the macropinocytosis inhibitor amiloride, which resulted in a more than 80% reduction in uptake (Figure S14).^43–45^ The observed perinuclear localization aligns with typical behaviors of synthetic delivery systems and many viruses,^46,47^ as most acidic endolysosomal vesicles accumulate in this region.^48,49^

While differences in uptake efficiency between the **SHS1** and **SHS2** particles were expected, the extent of these differences was striking. In two different cell lines (SH-SY5Y neuroblastoma and HEK cells), **SHS1** particles were taken up readily, while the **SHS2** particles were not (Figure 2C). Moreover, SH-SY5Y cells actively migrated toward the **SHS1** particles, capturing them via lamellipodia-like extensions, as shown in Figure 3A-B (see also Figures S18, S19 and Movie S1).^50^

**Figure 3:**
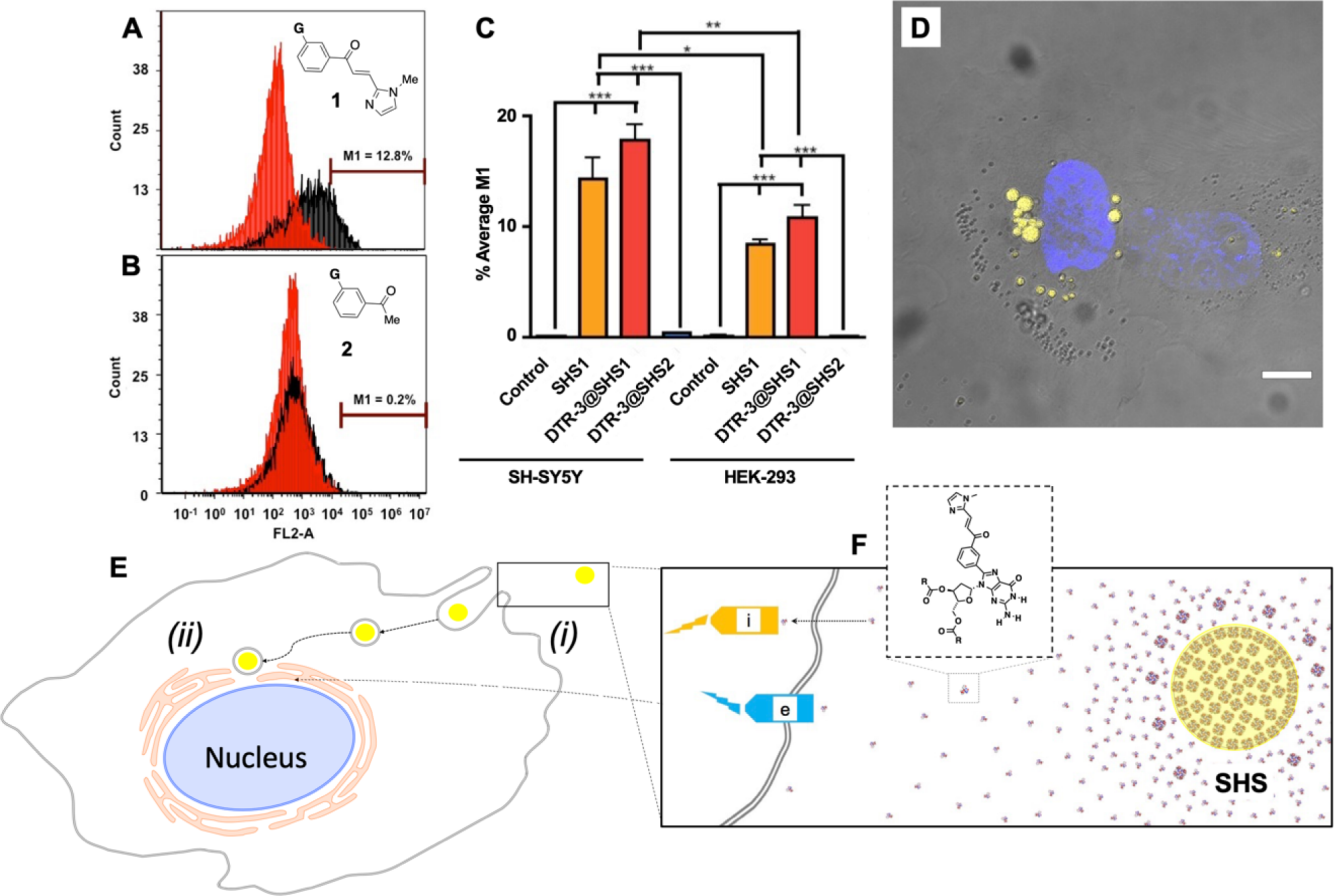
Cellular uptake of SHS particles depends on the structure of the constituent G-derivative but is relatively insensitive to molecular cargo. (A, B) Flow cytometry data showing SHS particle uptake in SH-SY5Y cells, highlighting that uptake is dependent on the G-derivative constituents. (C) Comparison of SHS uptake between SH-SY5Y and HEK-293 cells, with statistically significant differences shown (* *p* < 0.05; ** *p* < 0.01; *** *p* < 0.001). (D) Fluorescence image of SH-SY5Y cells after SHS particle internalization (scale bar = 5 μm), where nonfluorescent structures correspond to cellular vesicles and other cell bodies (see Figures S18, S19 and Movie S1 for more details). (E) Schematic representation illustrating (i) the uptake of SHS particles and (ii) their subsequent trafficking to the perinuclear region. (F) Proposed mechanism of **SHS1** uptake: the gradual dissolution of SHS particles generates a concentration gradient of compound **1**, which is recognized by putative extracellular (e) or intracellular (i) receptors, thereby triggering macropinocytosis.

Cellular uptake and trafficking of particles are known to depend on various factors, including size, shape, surface patterns,^24^ surface roughness, and chemical composition.^27,46^ Given the similarities between **SHS1** and **SHS2** in size and zeta potential (Table S1), one possible explanation for this specificity is that **SHS1** or its constituent compound (**1**) is recognized by an as-yet unidentified cell receptor,^51^ which may subsequently trigger macropinocytosis (Figure 3E-F). We hypothesize that the slow dissociation of **1** from **SHS1** creates a concentration gradient, guiding chemotaxis and promoting lamellipodia formation, leading to the observed uptake (Figure 3E).^52,53^

Although macropinocytosis is not directly regulated by specific cargo, as phagocytosis is, it can be induced by various stimuli, including drugs such as methamphetamine,^54^ nucleotides like cAMP,^55^ growth factors,^45,56^ and even nucleolin-mediated stimulation by anticancer G-quadruplex-containing aptamers.^57^ Furthermore, several cell receptors involved in the regulation of the innate immune response, as discussed later, have been reported to recognize G-derivatives.^58,59^ These findings suggest that the interaction between **SHS1** and specific cellular receptors may play a critical role in its selective uptake and intracellular trafficking.

### 3.4 Drug Delivery and Controlled Release of DOX in Cell Culture

Having established that SHS particles can be taken up by cells in vitro, we next tested their potential for delivering a biologically active small molecule. Specifically, we encapsulated and delivered the anticancer drug **DOX**, which serves as both a therapeutic agent and a model small-molecule probe with well-characterized biological activity and intracellular distribution. Earlier studies have shown that **DOX** interacts with SGQs^60^ and that SHS particles are suitable for its encapsulation.^34,40^ The results presented here further confirm that **DOX@SHS1** complexes provide an effective platform for the delivery and controlled release of **DOX** (Figure 4C).

**Figure 4:**
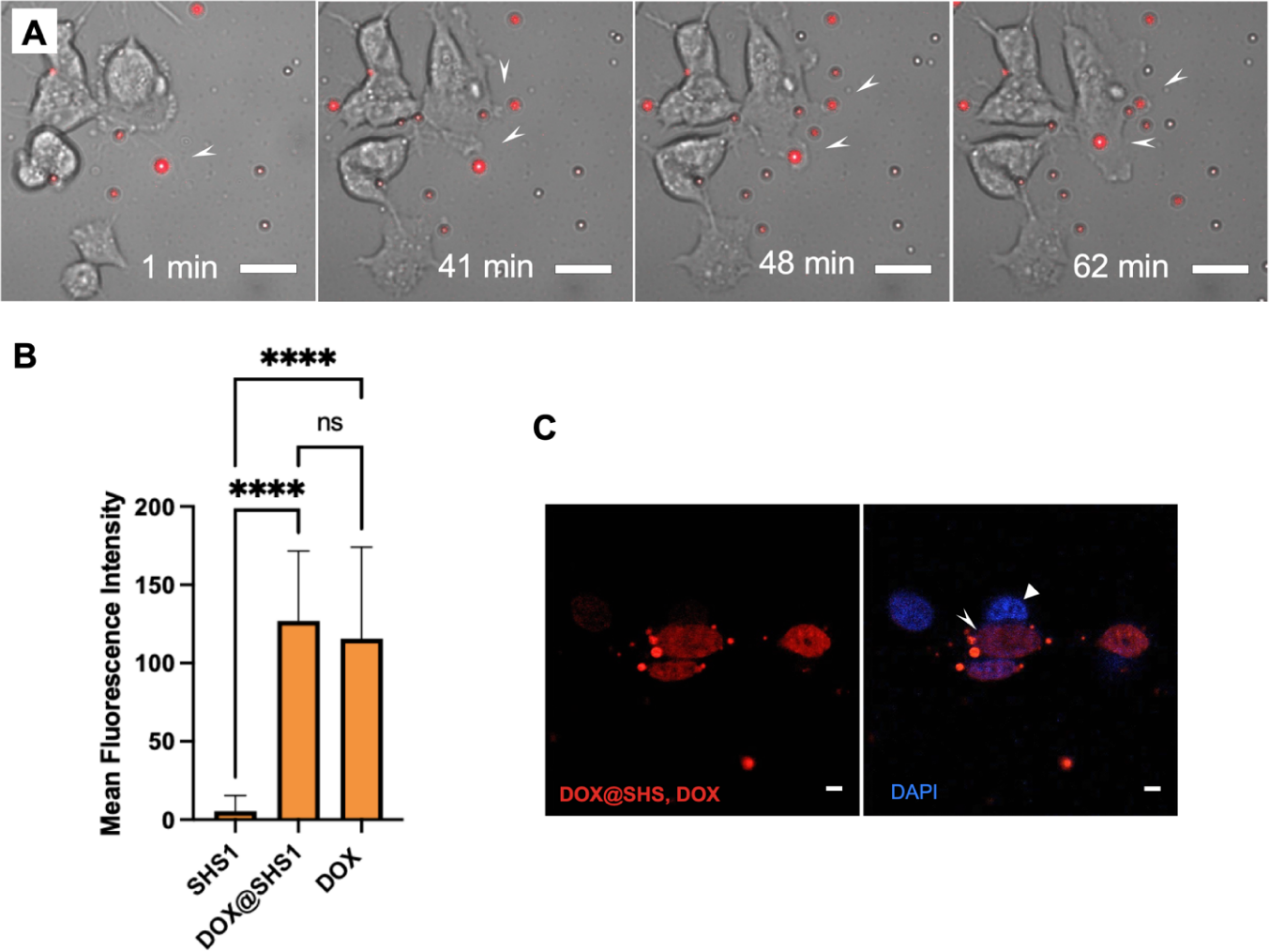
Intracellular delivery and cytotoxicity of **DOX@SHS1** complexes in neuroblastoma (SH-SY5Y) cells. (A) Live-cell imaging frames showing uptake of **SHS1** particles by SH-SY5Y cells (see Figures S17–S20, Movies S1 and S2 for additional details). (B) Mean Fluorescence Intensity (MFI) at 561 nm in cells treated with **SHS1** alone (control), **DOX@SHS1** (loaded with 100 equiv of **DOX**), and free **DOX** (100 equiv). (C) CLSM confirms intracellular delivery and nuclear accumulation of **DOX** following treatment with **DOX@SHS1**. After 12 h of incubation, only cells that internalized **DOX@SHS1** exhibited nuclear **DOX** fluorescence. The white arrowhead marks a nucleus where **DOX** (red) and **DAPI** (blue) colocalize, indicating successful intracellular drug release, while the triangle highlights a nucleus with no detectable **DOX** signal. Red fluorescence (561 nm) was used to visualize **SHS1**, **DOX@SHS1**, and **DOX**, and blue fluorescence (405 nm) was used for **DAPI**-stained nuclei. Scale bars: 20 μm (A) and 10 μm (C).

Prior to conducting cell culture experiments, we evaluated the pH-dependent release of **DOX** from **DOX@SHS1** by incubating the colloidal suspension at pH 7.2 and pH 5.0 (Figures S15 and S16). After 24 h, the amount of **DOX** released at pH 5.0 was nearly twice that released at pH 7.2, indicating enhanced release under acidic conditions. We attribute this behavior to the protonation of imidazole moieties within the G-derivatives at lower pH, which leads to an accumulation of counteranions within the SHS particles. This ionic buildup generates electrostatic repulsion between the SGQ subunits, causing the SHS to swell and expand. The resulting increase in particle volume and internal hydration facilitates faster diffusion of **DOX** into the surrounding medium (Figure S16B). This mechanism is analogous to previously reported behavior of polyhistidine and other imidazole-functionalized polymers used in drug and gene delivery, where protonation of the imidazole group induces backbone repulsion, polymer expansion, and enhanced cargo release.^61^

To distinguish the specific effects of encapsulated **DOX** from those of free drug in solution, control experiments included extensive PBS washing of the **DOX@SHS1** colloidal suspensions to remove unencapsulated **DOX** (see Supporting Information for details). CLSM imaging at 24 h revealed a reduction in the number of adherent **DOX@SHS1**-containing cells and a corresponding increase in detached, floating cells, consistent with drug-induced cytotoxicity. Quantitative MFI analysis (Figure 4B) shows that both **DOX@SHS1** and free **DOX** produce significantly higher nuclear fluorescence than the **SHS1** control, confirming effective delivery. However, the levels of nuclear **DOX** fluorescence were comparable between the **DOX@SHS1** and free **DOX** treatments, suggesting similar overall cellular exposure to the drug.

Importantly, CLSM live-cell imaging (Figure 4C and Movie S1) demonstrated that only cells that visibly internalized **DOX@SHS1** showed substantial **DOX** accumulation in the nucleus. In contrast, adjacent cells that did not internalize the particles exhibited little to no nuclear fluorescence. This observation strongly supports the conclusion that cytotoxicity is driven primarily by cellular uptake of **DOX@SHS1** rather than passive diffusion of **DOX** from the extracellular environment. While direct quantification of intracellular **DOX** concentration was not performed, the time-resolved fluorescence imaging approach used here provides a semiquantitative comparison of nuclear **DOX** accumulation between formulations. The enhanced intracellular release of **DOX** is likely triggered by the acidic conditions of the endolysosomal compartments, which facilitate drug dissociation from the SHS scaffold and promote effective nuclear delivery.

### 3.5 Gene Delivery Studies: Transfection Experiments Using pDNA@SHS1

Experiments using two different plasmid DNAs (**pGFP** and **pCri**) demonstrate that SHS particles serve as effective transfection agents, independent of gene sequence, and perform comparably to the well-established Lipofectamine 2000 system. We previously showed that **SHS1** particles can encapsulate both plasmids to form **pGFP@SHS1** and **pCri@SHS1** complexes.^34^ Confocal laser scanning microscopy (CLSM) confirmed that both complexes were readily taken up by SH-SY5Y neuroblastoma cells and enabled detectable protein expression after an 8-day incubation period (Figure 5). Control experiments using plasmid DNA without **SHS1**, or complexes with Lipofectamine, yielded no detectable fluorescence signal under identical conditions (Figures S21–S24), reinforcing the key role of **SHS1** in mediating successful transfection.

**Figure 5:**
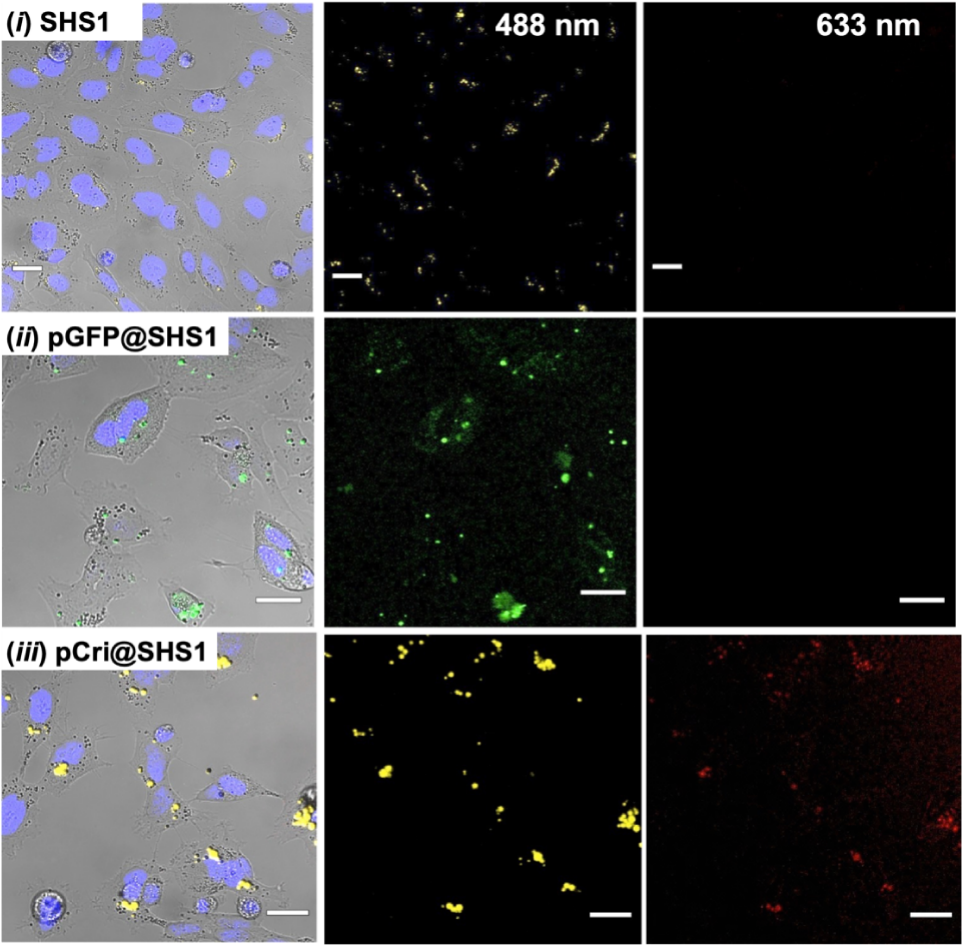
SHS particles as effective transfection agents for neuroblastoma cells. CLSM images show SH-SY5Y cells transfected with (i) **SHS1** alone, (ii) **pGFP@SHS1**, and (iii) **pCri@SHS1**. The cell nuclei were stained with Hoechst 33342 (blue) to facilitate visualization. Experimental details are provided in Figures S21–S25. Scale bars: 20 μm.

Quantitative analysis of Mean Fluorescence Intensity (MFI) revealed that **pGFP@SHS1** samples exhibited nearly double the fluorescence at 488 nm compared to **SHS1**-only controls (Figure S25A). However, because **SHS1** particles display intrinsic autofluorescence in this region, which overlaps with GFP emission, we also evaluated **pCri** transfection at 633 nm, a region where SHS1 does not fluoresce. These experiments confirmed the absence of **SHS1** autofluorescence at 633 nm, and **pCri@SHS1** samples showed a statistically significant 40-fold increase in MFI relative to controls. Notably, the MFI values for **pCri@SHS1** were slightly higher than those observed for **pCri** transfection with Lipofectamine, suggesting that **SHS1**-based delivery may offer improved efficiency in some contexts.

We hypothesize that the imidazole groups in the constituents of the **SHS1** particles (compound **1**) facilitate transfection by promoting endolysosomal escape, similar to the mechanism observed in imidazole-containing polymers.^62^ Interestingly, the distribution of expressed proteins differs between **pGFP@SHS1** and **pCri@SHS1** compared to Lipofectamine (Figures 5, S23 and S24). **SHS1**-mediated transfection results in more localized fluorescent regions, which colocalize with the internalized SHS particles (Figure 5). This phenomenon is more pronounced for **Cri** than for **GFP**, possibly due to GFP’s higher transfection efficiency, which enables broader diffusion throughout the cytoplasm. In contrast, under similar incubation conditions, Lipofectamine transfection leads to a more widespread cytoplasmic fluorescence distribution (Figure S23).

We propose that the colocalization of the expressed fluorescent proteins (FPs) is influenced by the spatial bias created by perinuclear localization, where endolysosomal escape, presumably aided by the “proton-sponge” effect,^23,63–65^ and subsequent nuclear import occur near the SHS particle. This interpretation aligns with the known heterogeneous distribution of nuclear pores,^66^ where “pore-free islands” could prevent the efficient export of FP-encoding mRNA.^67^ Additionally, SHS particles may act as scaffolds, concentrating the expressed FPs within their hydrogel-like interior. Additional future studies will be required to explore these hypotheses to better understand the mechanistic details underlying these observations.

Although this study focused on in vitro evaluation of SHS particles for drug and gene delivery, it builds on our prior work demonstrating their in vivo biocompatibility and immunomodulatory potential. In a previous mouse study, SHS particles coformulated with DNA plasmids significantly enhanced Th1-type cellular and humoral immune responses without observable toxicity.^36^ These findings provide important initial evidence supporting the safety and functional versatility of SHS particles in living systems, at least in the context of vaccine delivery. The current work extends these insights by investigating cellular uptake, intracellular trafficking, and delivery efficacy under controlled in vitro conditions.

While this study used Lipofectamine as a benchmark for gene delivery, broader comparisons to other systems are essential to contextualize the distinctive properties of SHS particles. Viral vectors offer high transfection efficiencies but are often limited by immunogenicity, cargo size restrictions, and high production costs. Lipid and polymer-based carriers, including Lipofectamine and polyethylenimine (PEI), are widely used but suffer from reproducibility issues and cytotoxicity concerns, particularly at effective doses.^68^ Inorganic carriers, while structurally versatile, frequently involve complex syntheses and limited biodegradability. In contrast, SHS particles are composed of simple guanosine derivatives and form through straightforward supramolecular self-assembly in aqueous solution without surfactants or organic solvents. This provides access to structurally modular, biocompatible carriers with tunable uptake properties. Furthermore, our use of Lipofectamine as a reference aligns with recent calls for standardized benchmarking in gene delivery research.^69^ As the field moves toward safer, reproducible, and modular nonviral platforms, we believe SHS particles represent a promising and underexplored alternative for nucleic acid delivery.

## 4 Conclusions

Supramolecular chemistry offers powerful strategies to address longstanding challenges in drug delivery and therapeutic development. However, realizing this potential requires a detailed understanding of how synthetic supramolecular systems behave within biological environments. The SHS particles presented in this study represent a unique class of hierarchical supramolecular assemblies with considerable promise as platforms for cellular and biomedical applications. They bridge the gap between polymer-based systems and small-molecule precision, leveraging the defined architecture of G-derivatives and their predictable self-assembly into colloidal structures. Their structure-dependent cellular uptake, combined with demonstrated biocompatibility within the tested concentration range, positions SHS particles as strong candidates for multifunctional use in biology. For example, the ability to encapsulate and release doxorubicin not only supports their application in anticancer drug delivery but also highlights their potential as model systems for small-molecule probe development.^70^ Additionally, the successful transfection of plasmids leading to fluorescent protein expression illustrates their broader utility in gene delivery and cellular engineering.^68^ These findings open the door to systematic structure–activity relationship studies, particularly when combined with our recently developed flow cytometry method for quantifying SHS encapsulation efficiency.^32,33^

Nevertheless, SHS particles currently face limitations when compared to well-established lipid and polymer-based platforms, especially regarding large-scale production, long-term stability, and delivery efficiency for complex cargos such as mRNA or CRISPR-associated components. While lipid nanoparticles have already demonstrated clinical utility, most notably in mRNA vaccines, SHS systems are still in the early stages of characterization and optimization. Continued progress will therefore require focused studies on the structure–function relationships governing SHS behavior, stability, and performance in increasingly complex biological contexts.^36^ These efforts are crucial to unlock their full potential as versatile and tunable delivery vehicles for next-generation biomedical applications. The growing need for effective and adaptable delivery systems, highlighted by the challenges of genome editing technologies,^71^ and the urgency of pandemic preparedness,^17^ underscores the importance of investing in innovative supramolecular platforms like SHS particles.

## Supplementary Material







## Notes

General experimental procedures; preparation and stabilization of SHS particles and DTR-3 encapsulation; zeta potential and SEM measurements; confocal microscopy studies of cellular uptake and trafficking, including Z-stack analysis, lysosomal colocalization, and macropinocytosis inhibition assays; flow cytometry data comparing uptake across cell lines; in vitro drug release studies and delivery of doxorubicin to neuroblastoma cells; plasmid preparation and gene delivery experiments using **SHS1** complexes; and fluorescent protein expression in SH-SY5Y cells following transfection with **pGFP** and **pCri** (PDF)
Live-cell imaging for the uptake of **DOX@SHS1** by SH-SY5Y cells­(MP4)
Live-cell imaging with stained nuclei for the uptake of **DOX@SHS1** by SH-SY5Y cells (MP4)
