# Supplementary material for: Guanosine-Based Supramolecular Particles for Enhanced Drug and Gene Delivery in Cell Culture
Source: ACS Appl Bio Mater. 2025 Jun 10;8(7):5625–33. doi: 10.1021/acsabm.5c00201 (PMC12284859; doi:10.1021/acsabm.5c00201)
Supplement: Supplementary file 1 [file mt5c00201_si_001.pdf]

SUPPORTING INFORMATION:

Guanosine-based supramolecular particles for enhanced drug and gene delivery in cell culture

Luis M. Negrón,[3] Edwin Vázquez-Rosa,[4] Luxene Belfleur,[1] Tanya L. Díaz,[1]

Bismark Madera-Soto,[4,2] Irving E. Vega,[5] and José M. Rivera*,[1,2]

1. Department of Chemistry, University of Puerto Rico at Río Piedras, San Juan, PR, 00925
2. Molecular Sciences Research Center, University of Puerto Rico, San Juan, PR 00926
3. Department of Chemistry, University of Puerto Rico at Cayey, Cayey, PR 00736
4. Department of Biology, University of Puerto Rico at Río Piedras, San Juan, PR, 00931
5. Department of Translational Science and Molecular Medicine, College of Human Medicine, Michigan State University, Grand Rapids, MI, 49503

*Corresponding E-mail: jose.rivera151@upr.edu

Table of Contents

General experimental procedures 2
A. SHS particles and their encapsulation complexes (Figs. S1-S3) 2
  A1. Self-assembly and fixing of SHS particles 2
  A2. Encapsulation procedure for **DTR-3@SHS1** 3
  A3. Scanning Electron Microscopy (SEM) 3
  A4. Zeta potential and SEM measurements 4
B. Cellular uptake & trafficking of SHS1 (Figs. S4-S14) 4
  B1. Confocal Laser Scanning Microscopy Studies 5
  B2. Z-Stack studies (CLSM) 9
  B3. Flow Cytometry (FC) Studies 10
  B4. Trafficking studies 14
  B5. Macropinocytosis inhibitor (amiloride) studies 15
C. Drug delivery & controlled release (Figs. S15-S20) 16
  C1. In vitro controlled release experiments 16
  C2. Drug delivery to neuroblastoma cells 18
D. Gene delivery in vitro (Figs. S21-S25) 21
  D1. General information of plasmid preparation 21
  D2. SH-SY5Y cells incubation with **SHS1** 21
E. Supporting References 27

General experimental procedures

A. SHS particles and their encapsulation complexes (Figs. S1-S3)

A1. Self-assembly and fixing of SHS particles

The SGQs were prepared from 8ArG derivatives **1** or **2** (5 mM; 650 µL) in 1X PBS (pH 7.4; Fisher Scientific) having a concentration of KI of 2 M for **1** and 700 mM for **2**. The resulting solutions were then stored overnight (~12 h) in the refrigerator (-10 °C) without further treatment. The formation of the SHS colloid from the corresponding SGQ was accomplished by triggering the LCST via heating the solution in a water bath at 40 °C. After formation, the SHS particles were kinetically stabilized or "fixed" by diluting 0.1 mL of the SHS colloid in PBS (1.57 mL; 1X at pH 7.4), which resulted in a solution of SHS particles (0.303 mM) having a new concentration of KI of 121 mM for **1** and 36.4 mM for **2**. The nomenclature **GUEST@SHS1/2** indicates the complexes between a given guest molecule (e.g., **DOX**, **DTR-3**) and the **SHS1** or **SHS2**, respectively.[1–3]

As described in the main article and this supporting information the terms **SHS1** and **SHS2**, to refer to those made from 8ArG derivatives **1** and **2**, respectively. The kinetically stable SHS particles are suitable for a variety of physical manipulations (e.g., dilutions) while preserving their integrity and shape without the need for further covalent crosslinking. The reported concentrations of SGQs, and SHS particles refer to the amount of the 8ArG derivatives (**1** (MW = 1,072.07 Da) or **2** (MW = 979.99 Da)) used to make each supramolecular species.

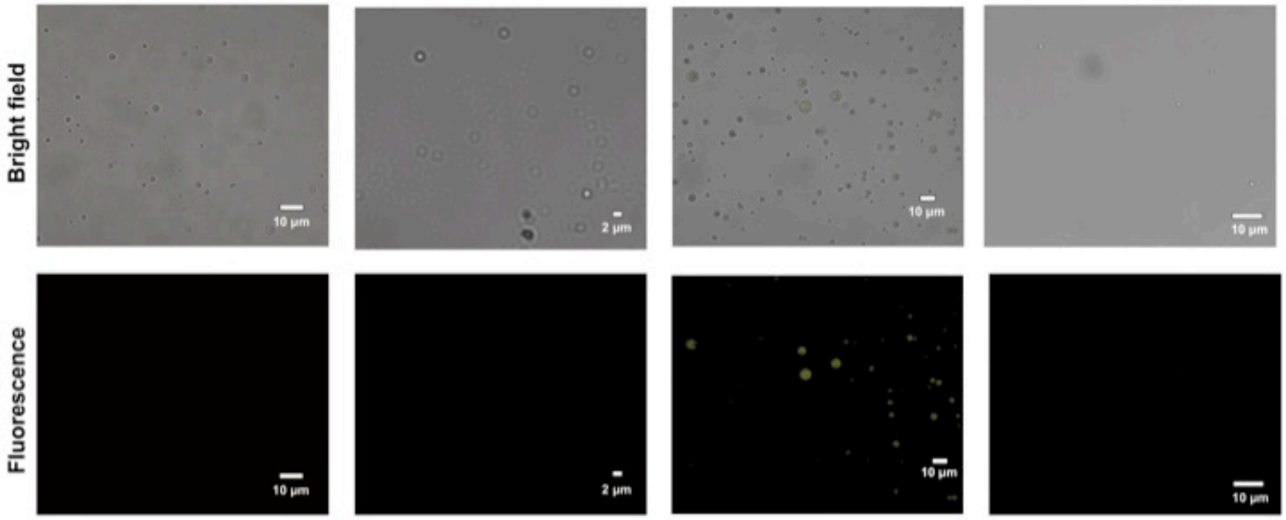

***Figure S1**. SHS particles prepared from compounds **1** and **2** in PBS and cell culture media. Columns 1 and 2 show **SHS1** and **SHS2** in PBS, while columns 3 and 4 show the same particles in Dulbecco's Modified Eagle Medium/Nutrient Mixture F-12 (DMEM F-12) supplemented with 10% fetal bovine serum (FBS). The observed fluorescence of **SHS1** in DMEM F-12 (panel c) may result from encapsulation (i.e., concentration) of phenol red, riboflavin, or other fluorescent components present in the medium. All samples were prepared at pH 7.4 and 25 °C: **SHS1** (27.5 µM; 11 mM KI) and **SHS2** (50.5 µM; 6.1 mM KI). Images were acquired using an excitation wavelength of 561 nm and an emission filter with a band pass of 575–615 nm (IR).*

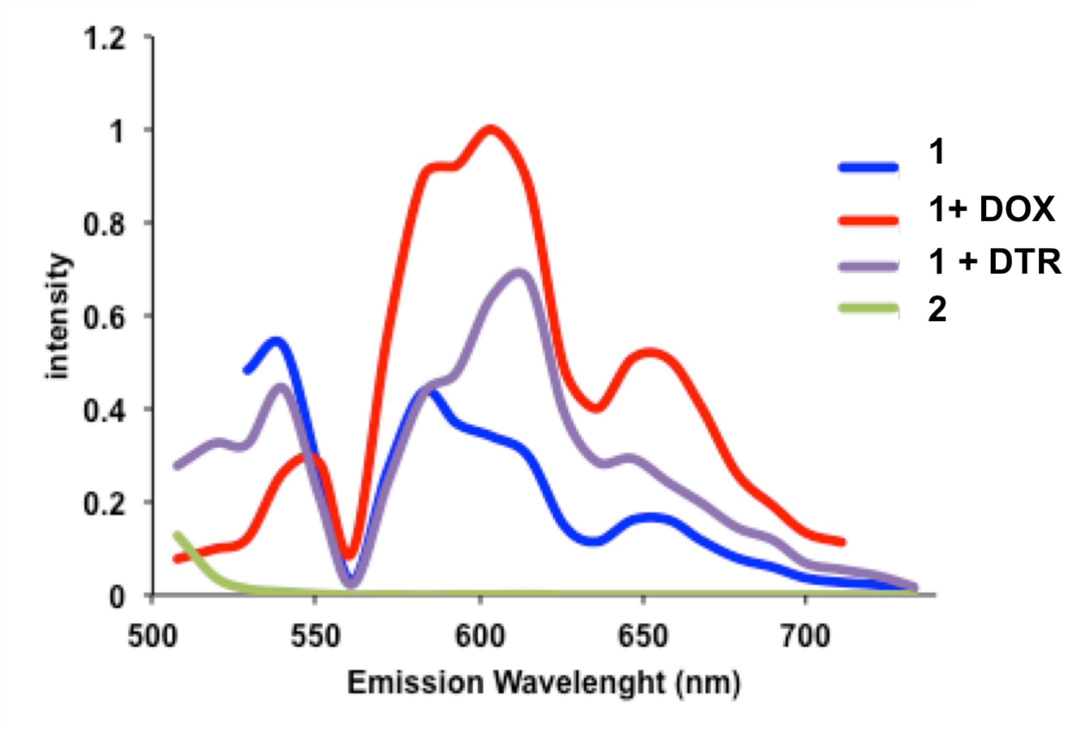

Figure S2. *Emission pattern of **SHS1**, **DOX@SHS1**, **DTR-3@SHS1**, **SHS2** (λ_{ex} = 488 nm, pH 7.20 ± 0.01, 25 °C, 303 μM of both **SHS1** and **SHS2**, 121 mM KI and 36.4 mM KI, respectively, 100 equiv DOX and 20.5 equiv of Dextran Texas Red 3 kDa (DTR-3)).*

*A2. Encapsulation procedure for **DTR-3@SHS1***

A volume of 100 μL of SHS colloid formed as previously described in section B1, was incubated with **DTR-3** (423 μM; 20.5 equiv) by stirring for 3 min at 40 °C (the same procedure was performed with 3.1 equiv **DTR-3** (423 μM) used for Flow Cytometry experiments described in part C). The resulting **DTR-3** concentrations after diluting the 20.5 and 3.1 equiv. **DTR-3** in SHS colloid (containing PBS) were 171 μM and 25.6 μM, respectively. Next, the **DTR-3@SHS1** was washed with PBS to remove excess of **DTR-3** using a sedimentation cone and fixed following procedure described in section B to obtain a final concentration of **DTR-3@SHS1** (303 μM). Finally, an aliquot (100 μL) of **DTR-3@SHS1** (or **DTR-3@SHS2**) was added to the cell culture medium (1.0 mL) used to incubate the SH-SY5Y cells.

A3. Scanning Electron Microscopy (SEM)

A high-resolution field emission JEOL JSM-7500F Scanning Electron Microscope was used to characterize selected samples as previously described by us.[1] After preparation of the samples as described in Section B, they were deposited on the copper SEM grids by placing a drop on each grid using a Pasteur pipette (drop-casting method). Each grid was held by non-magnetic tweezers located over a heat source (hot plate) at 36 °C to allow for air-drying of the solvent. The resulting dry sample was analyzed in ultrathin carbon film/holey carbon 400 mesh cooper grids without coating the solids.

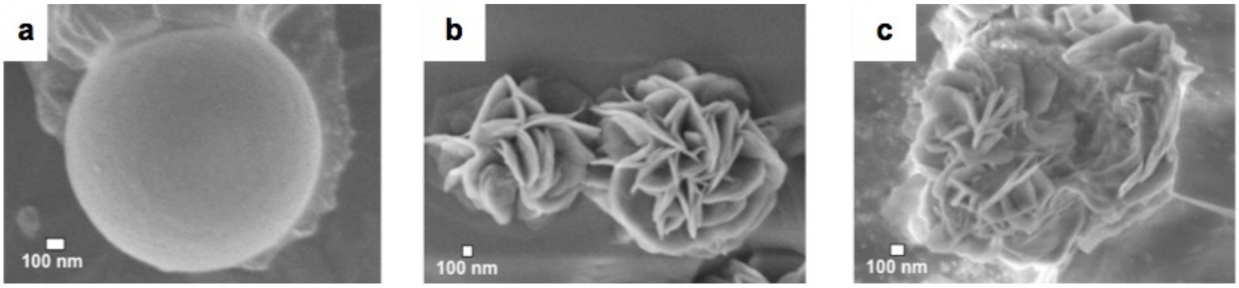

Figure S3. *SEM images of: (a)* ***SHS1****, (b)* ***pCri@SHS1****, and (c)* ***pGFP@SHS1****.*[1,4–6] *Adapted with permission from ref. 6: Negrón, L. M.; Díaz, T. L.; Ortiz-Quiles, E. O.; Dieppa-Matos, D.; Madera-Soto, B.; Rivera, J. M. Langmuir* ***2016****, 32, 2283–2290. Copyright 2016 American Chemical Society.*

<u>A4. Zeta potential and SEM measurements</u>

Zeta potentials (ZP) were measured with Zetasizer Nano ZS (model ZEN3600 from Malvern Instruments Ltd.) with the parameters described in Section A1-2. The samples were measured in disposable folded capillary cells (DTS1070) from Malvern Instruments Ltd. The results were extracted from Malvern Zetasizer Software version 7.10 to construct the graphs using Excel and GraphPad PRISM version 5.0.

Table S1. *Zeta potentials of SHS particles, corresponding complexes* ***GUEST@SHS1****, and guests after incubating for 1 h (0.303 mM in* ***1****, 121 mM KI, 1X PBS, pH 7.4).*

Sample	ZP (mV)
**DOX**	+9.5 ± 0.3
**DTR-3**	-6.5 ± 1.6
**pCri**	-32.9 ± 2.1
**pGFP**	-34.6 ± 2.4
**SHS1**	-12.7 ± 0.3
**SHS2**	-3.3 ± 0.7
**SHS1** (in Cell Media)	-9.6 ±0.8
**SHS2** (in Cell Media)	-8.6 ±1.0
**DTR-3@SHS1**	-5.9 ± 0.2
**DOX@SHS1**	+6.9 ± 0.7
**pCri@SHS1** (Crimson)	-9.4 ± 0.3
**pGFP@SHS1**	-8.5 ± 0.4

B. Cellular uptake & trafficking of SHS1 (Figs. S4-S14)

For internalization and encapsulation experiments we used a Zeiss LSM 510 Meta Confocal Laser Scanning Microscope (CLSM) with an excitation range of 514-515 nm and an emission range of 565-615 nm. After incubating the cells with **SHS1** and its **Guest@SHS1** complexes, the samples were fixed with fluorescence-mounting medium (Dako, Inc.) in two well-cubed coverslip chambers (Fisher Scientific). All the CLSM images and movies were processed with Zeiss LSM Image Browser software.

B1. Confocal Laser Scanning Microscopy Studies

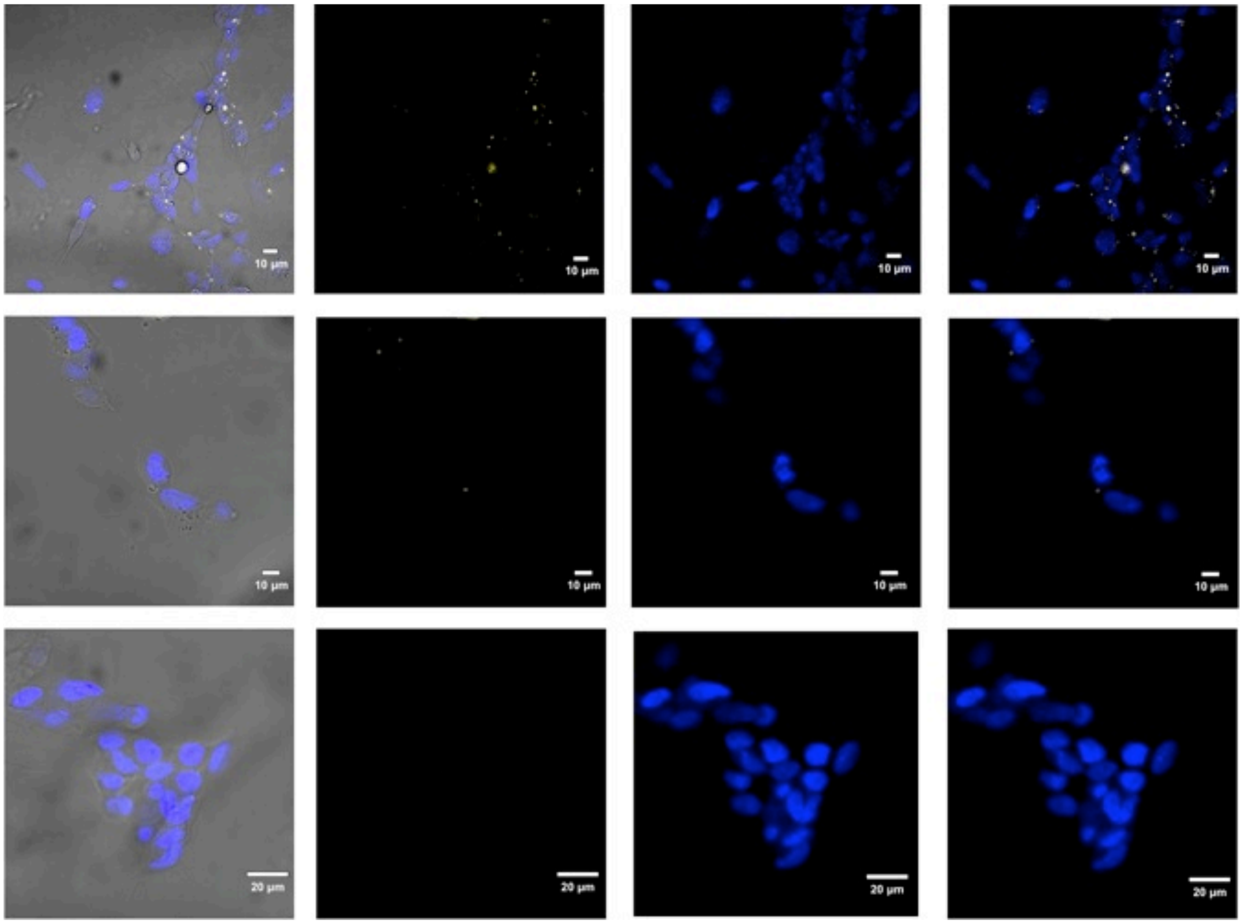

Figure S4. *Uptake microscopy studies of SHS particles in SH-SY5Y cells. Rows correspond to: (top)* ***SHS1****, (center)* ***SHS2****, (bottom) no particles (control). Columns correspond to: (1) Bright field merged, (2) the yellow channel (561 nm) for the* ***SHS1*** *particles, (3) the blue channel (405 nm) for the DAPI nuclear stain, and (4) fluorescence merged. The emission filters used were BP 575-615 IR (λ_{ex} = 561 nm) and BP 420-480 (λ_{ex} = 405 nm). Cells were incubated for 12 h in presence of* ***SHS1*** *(282 µM; 38.4 mM KI) or* ***SHS2*** *(50.5 µM; 6.1 mM KI). The* ***SHS2*** *and the control samples show no significant fluorescence under similar conditions (λ_{ex} = 561 nm; emission filter: BP 588-738).*

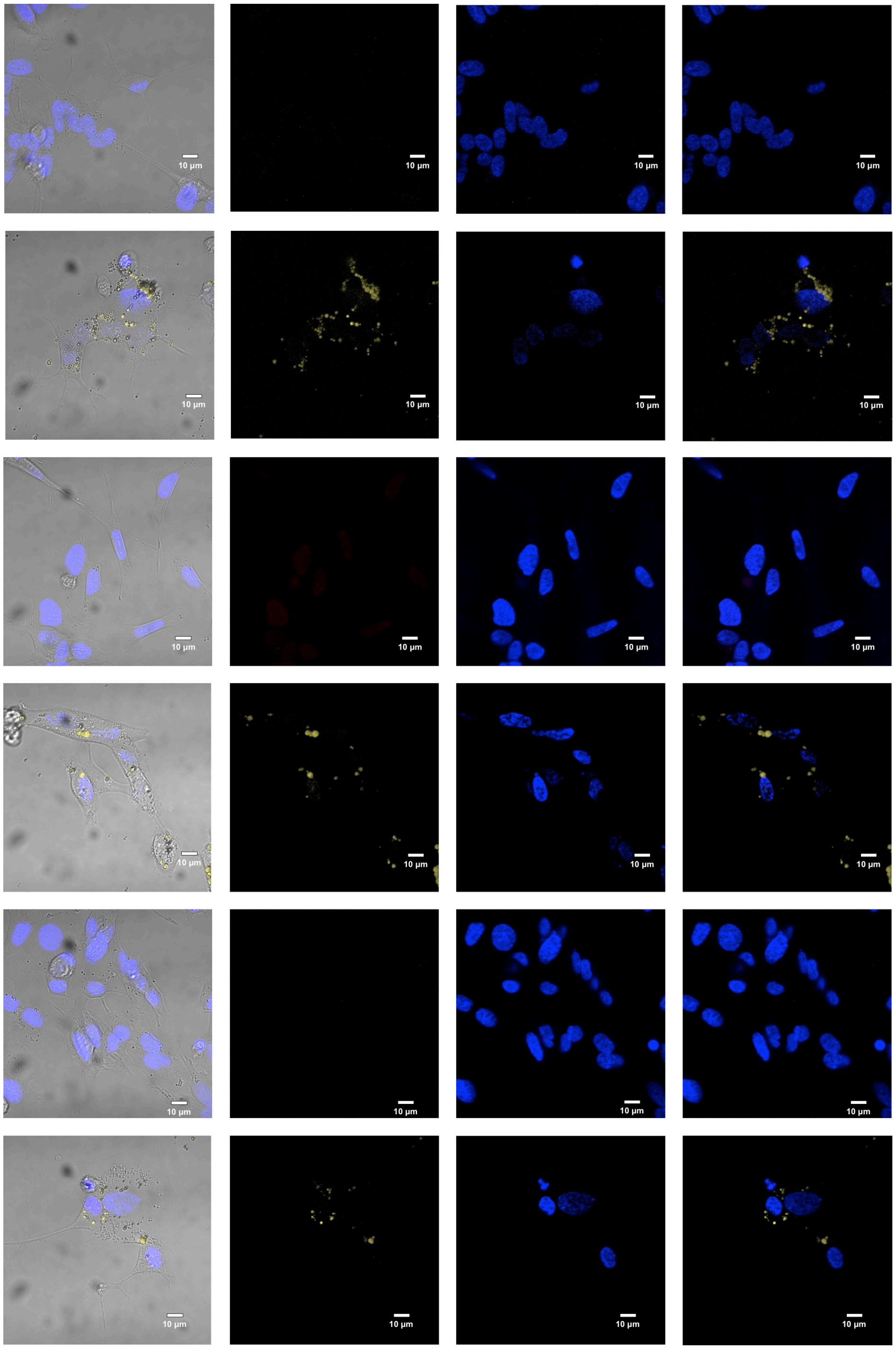

Figure S5. *Uptake of **SHS1** particles in SH-SY5Y cells as a function of time. Rows correspond to: (1-2) 2 days, (3-4) 5 days, (5-6) 8 days, with even rows (2, 4, 6) having **SHS1** particles (69.9 µM with 27.9 mM KI) and odd rows (1, 3, 5) being control images without particles. The columns correspond to: (1) Bright field merged, (2) the yellow channel (561 nm) for the **SHS1** particles, (3) the blue channel (405 nm) for the Hoechst nuclear stain, and (4) fluorescence merged. The emission filters used were BP 575-615 IR (λ_{ex} = 561 nm) and BP 420-480 (λ_{ex} = 405 nm).] Some images were obtained with long pass (LP) filters instead BP filters. The rows of images are enumerated as 1-6 from top to bottom.*

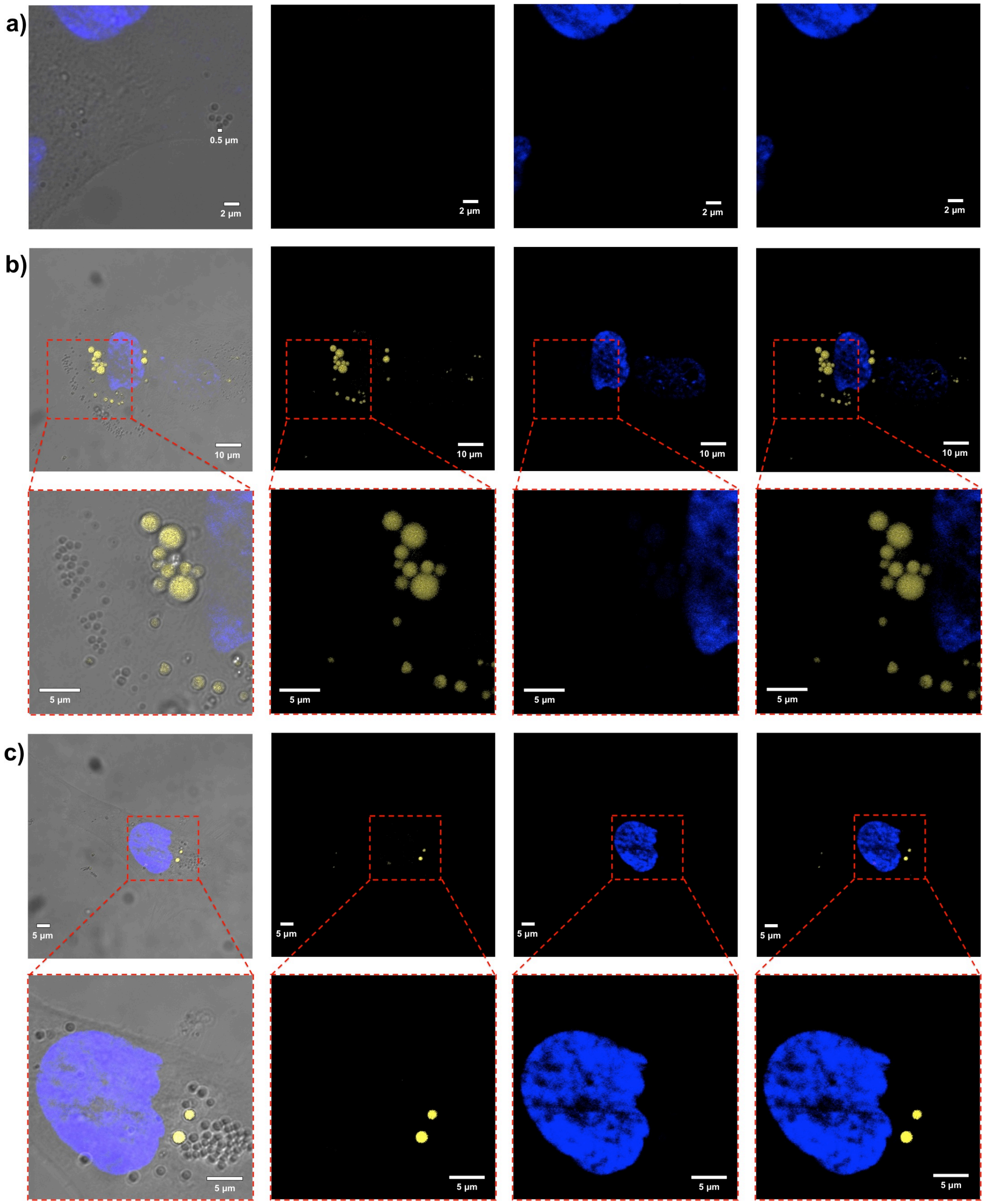

Figure S6. *Additional images of SH-SY5Y cells after incubating for 8 days (a) without* ***SHS1*** *(control) and (b-c) with* ***SHS1*** *particles. Columns correspond to: (1) Bright field merged, (2) the yellow channel (561 nm) for the* ***SHS1*** *particles (69.9 µM with 27.9 mM KI), (3) the blue channel (405 nm) for the Hoechst nuclear stain, and (4) fluorescence merged. Images b and c are illustrate that perinuclear localization is reproducible, and independent on the apparent uptake efficiency (high, for b and low for c). The emission filters used were BP 575-615 IR (λ_{ex} = 561 nm) and BP 420-480 (λ_{ex} = 405 nm).*

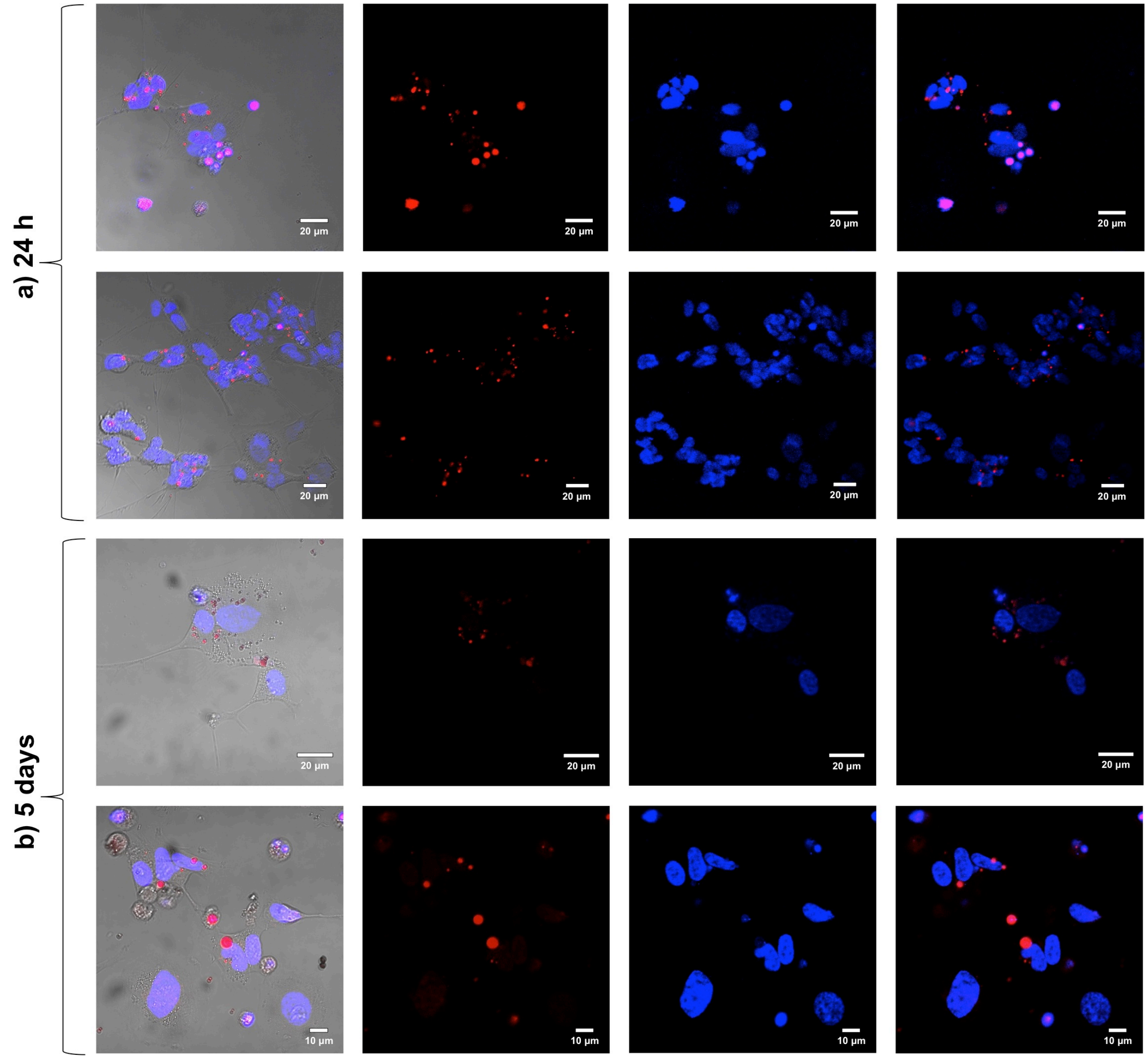

Figure S7. *Uptake of* ***DTR-3@SHS1*** *in SH-SY5Y cells after incubating for (a) 24 hr and (b) 5 days. Details: a)* ***DTR-3@SHS1*** *after 24 h of incubation (****SHS1*** *(27.5 μM) with KI (11.0 mM),* ***DTR-3*** *(20.5 equiv)); b)* ***DTR-3@SHS1*** *after 5 days of incubation (****SHS1*** *(69.9 μM) with KI (27.9 mM), DTR-3 (3.1 equiv)). Columns correspond to: (1) Bright field merged, (2) the 561 nm channel for the* ***DTR-3@SHS1*** *complexes, (3) the blue channel (405 nm) for the Hoechst nuclear stain, and (4) fluorescence merged. The emission filters used were BP 575-615 IR (λ_{ex} = 561 nm) and BP 420-480 (λ_{ex} = 405 nm).*

B2. Z-Stack studies (CLSM)

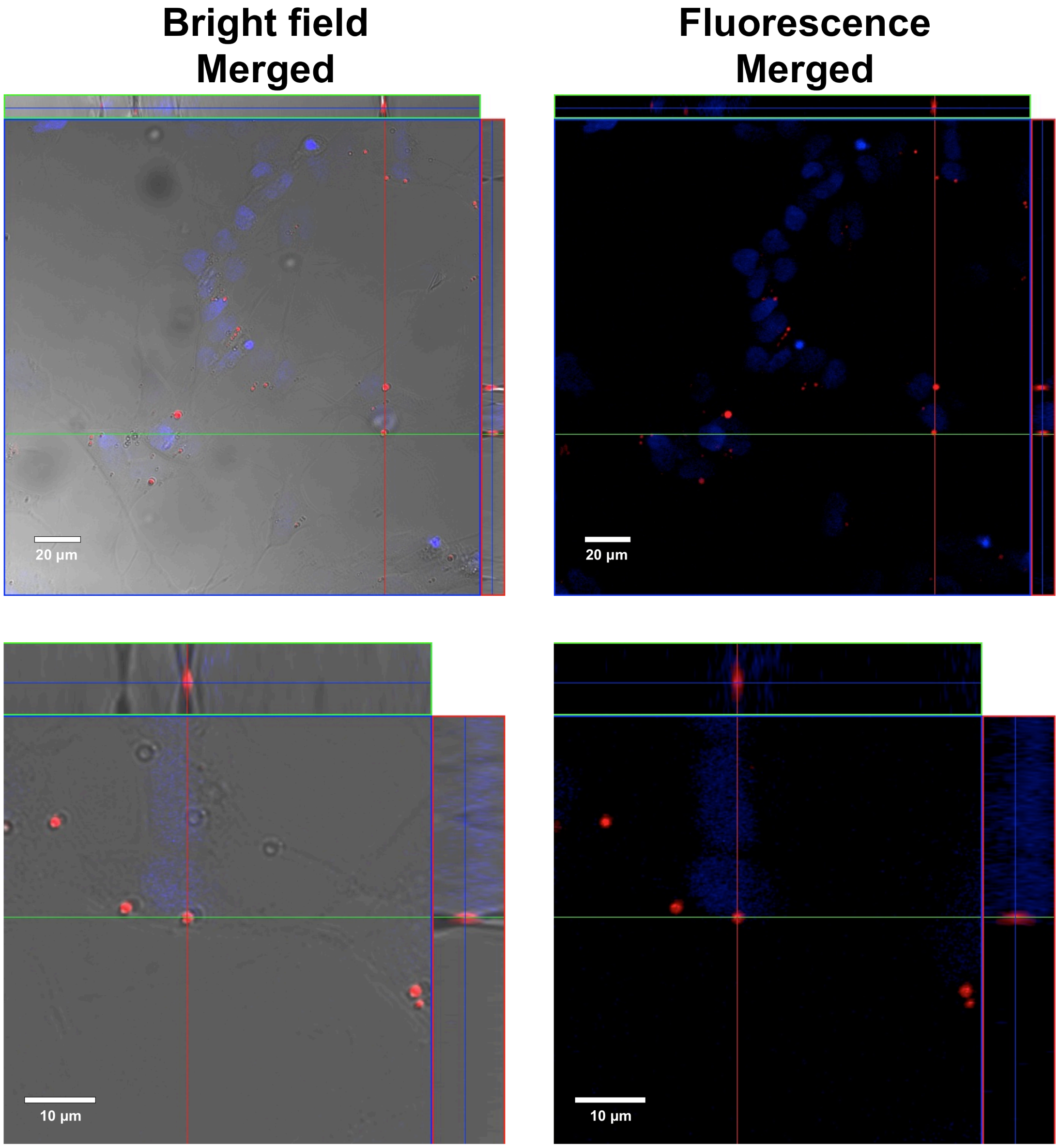

Figure S8. *Z-stack ortho images of* ***DTR-3@SHS1*** *in SH-SY5Y cells after 24 hr incubation. The two sets of representative images shown confirm the intracellular localization after 24 h incubation. The emission filters used were LP 575 (λ_{ex} = 561 nm) and BP 420-480 (λ_{ex} = 405 nm). Concentrations:* ***SHS1*** *(27.5 µM; with KI (11.0 mM)) and DTR-3 (20.5 equiv).*

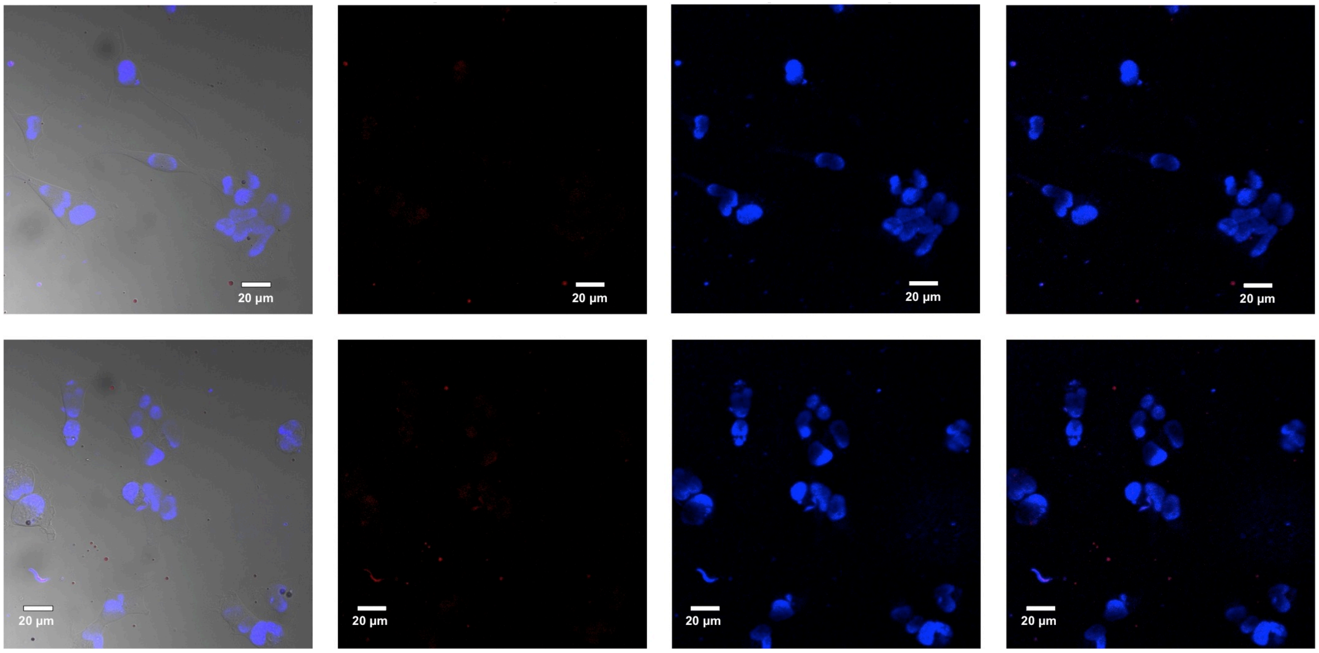

Figure S9. *Images of* ***DTR-3@SHS1*** *in HEK-293 cells after 5 hr incubation. Rows correspond to two sets of representative images. Columns correspond to: (1) Bright field merged, (2) the red channel (561 nm) for the* ***SHS1*** *particles, (3) the blue channel (405 nm) for the DAPI nuclear stain, and (4) fluorescence merged. The emission filters used were LP 575 (λ_{ex} = 561 nm) and BP 420-480 (λ_{ex} = 405 nm). Concentrations:* ***SHS1*** *(27.5 µM; with KI (11.0 mM)) and* ***DTR-3*** *(20.5 equiv).*

<u>B3. Flow Cytometry (FC) Studies</u>

The samples to perform flow cytometry analysis were prepared as follows: **SHS1**, **SHS2**, **DTR-3@SHS1** and **DTR-3@SHS2** following the preparation protocol described earlier (Section B). Then we added 100 µL to a 1.0 mL well containing SH-SY5Y human SH-SY5Y cells immersed in Dulbeco's Modified Eagle's Medium/Nutrient Mixture F-12 (DMEM F-12 from Sigma) after incubation for 12 h. Cells were seeded at 1 x 10^5 cells/well in 48-well plates (1.0 mL). Further treatment to perform flow cytometry analysis consisted in removal of the cell medium from the cultured cells described previously and replace it with fresh cell medium with 0.25% trypsin for 10 min to remove the cells from the plate. The mixture was then transferred to a 1.0 mL Eppendorf tube and then centrifuged (1000 rpm) for 10 min. The supernatant was removed and replaced with PBS (250 µL) to homogenize the cells and analyzed with a BD Acurri C6 flow cytometer.

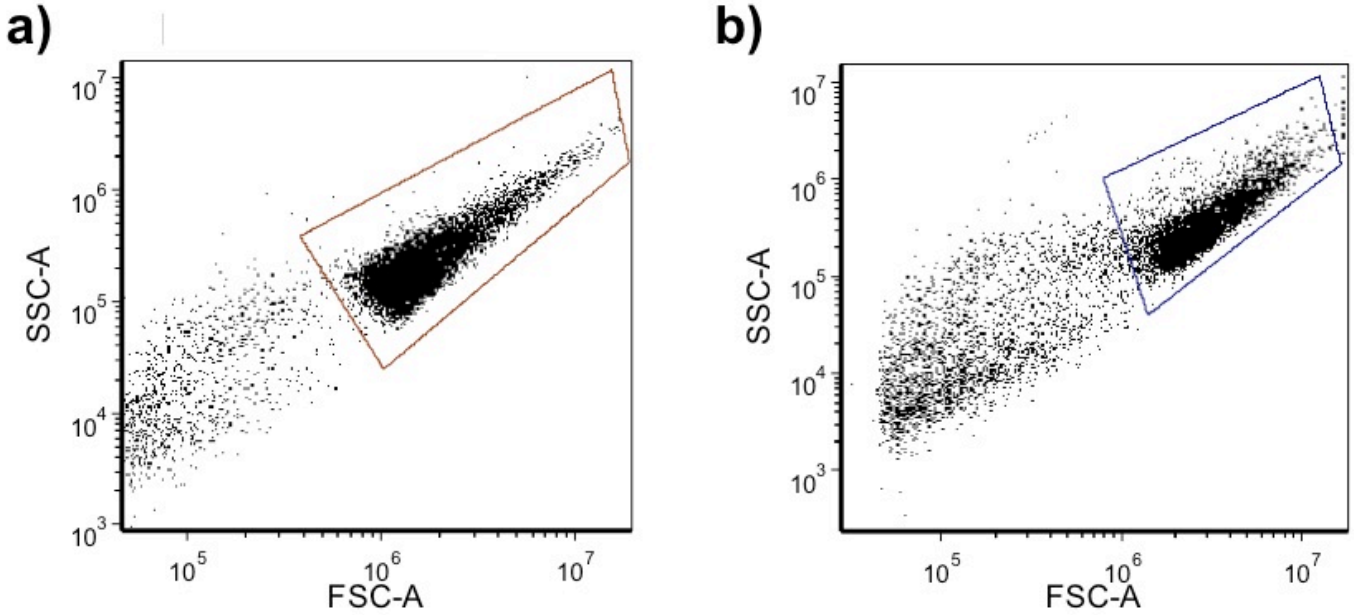

Figure S10. *Scatter dot plots from FC analysis of SH-SY5Y (a) and HEK-293 (b) cells incubated with* ***SHS1****. Plots represent measurements of* 10^4 *events collected after incubating the cells for 12 h (see Figure S11 for further details; morphologic gate of 9027 events) in DMEM F-12 supplemented with 10% fetal bovine serum (FBS) media (morphologic gate of 7279 events). Morphologic gates were used as a control to obtain percent of gated cells and the median fluorescence values reported.*

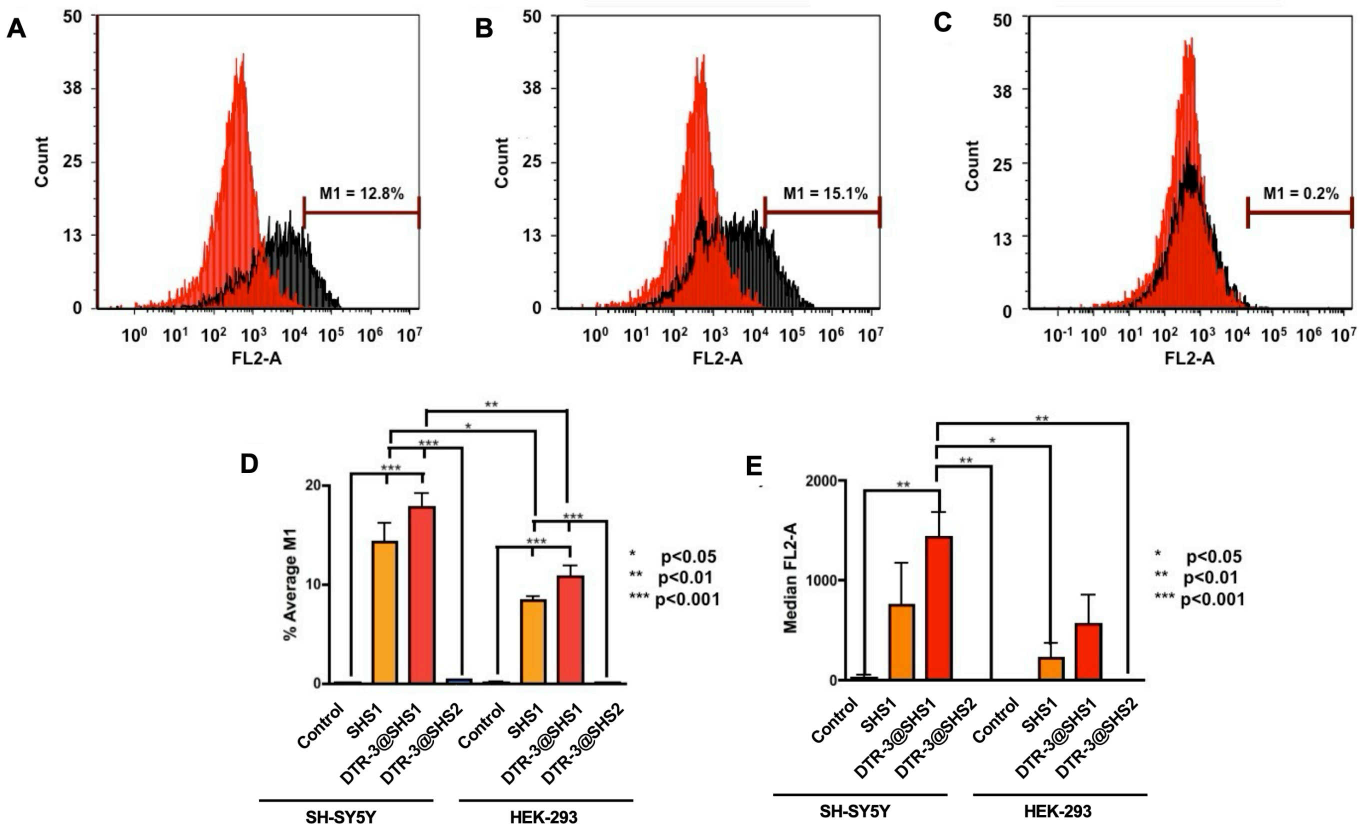

Figure S11. *Histogram plots for the cellular uptake of SHS particles by SH-SY5Y and HEK-293 cells. Negative controls (red histograms) using in SH-SY5Y neuroblastoma cells alone and after the incubation (black histogram) with: A)* ***SHS1****, B)* ***DTR-3@SHS1****, C)* ***DTR-3@SHS2****; (D, E) Bar graphs obtained from: (d) the average M1 percentage (n = 3), and (e) Median FL2-A (n = 3) compared in both cases to the positive response of both types of cells against the treatments used in the histograms shown in A, B, and C. The M1 percentage is reported from the shift in red fluorescence with respect to the control, which is the SH-SY5Y cells alone (red control histogram) against a positive response of the different treatments (black histogram) in A, B, and C conditions after incubating for 12 h with* ***SHS1*** *and* ***SHS2*** *(both at 27.5 µM), with KI (11.0 mM for* ***SHS1*** *and 3.31 mM for* ***SHS2****) and* ***DTR-3*** *(3.1 equiv). The histograms correspond to the previously selected gated cells in the scatter dot plots shown in Figure S10.*

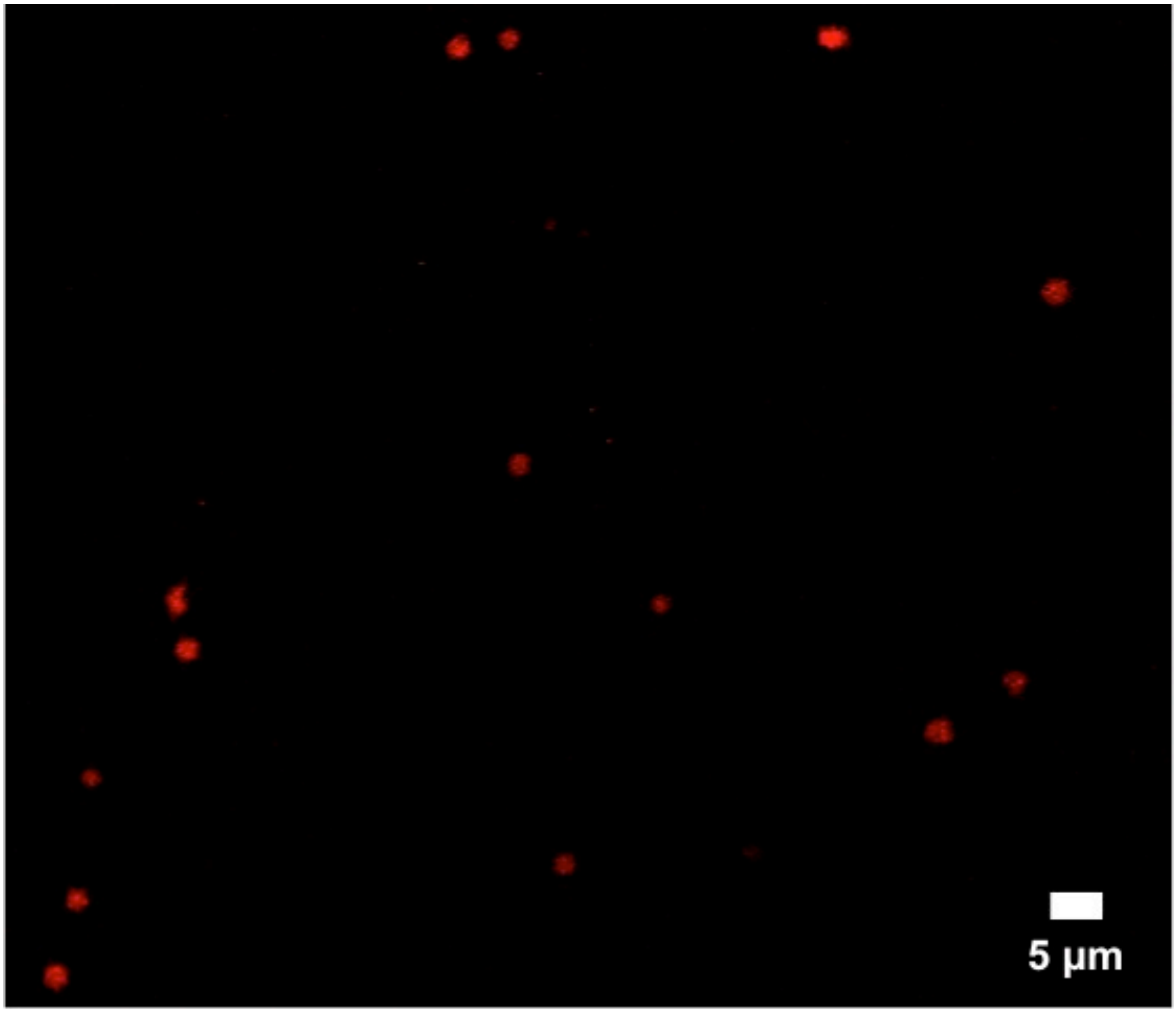

Figure S12. *CLSM image of* ***DTR-3@SHS2*** *used in cellular uptake experiments analyzed by FC (Figure S9-S10). Measured at* λ_{ex} *= 561 nm and emission filter: band pass (BP) 575-615 IR using PBS media at 25 °C, pH 7.4,* ***SHS2*** *(27.5 µM) with KI (3.31 mM),* ***DTR-3*** *(3.1 equiv).*

B4. Trafficking studies

Trafficking to endolysosomal compartments was evaluated using the pH sensitive (acidotropic) LysoTracker® Deep Red probe (referred as LysoTracker). Human neuroblastoma (SH-SY5Y) cells immersed in culture medium (1.0 mL) were incubated (3 days) with either the LysoTracker probe alone (Figure S11a) or in the presence of the *SHS* particles (300 µL) (Figure S11b). The yellow channel was designated for the 561 nm laser, while the red channel for 633 nm.

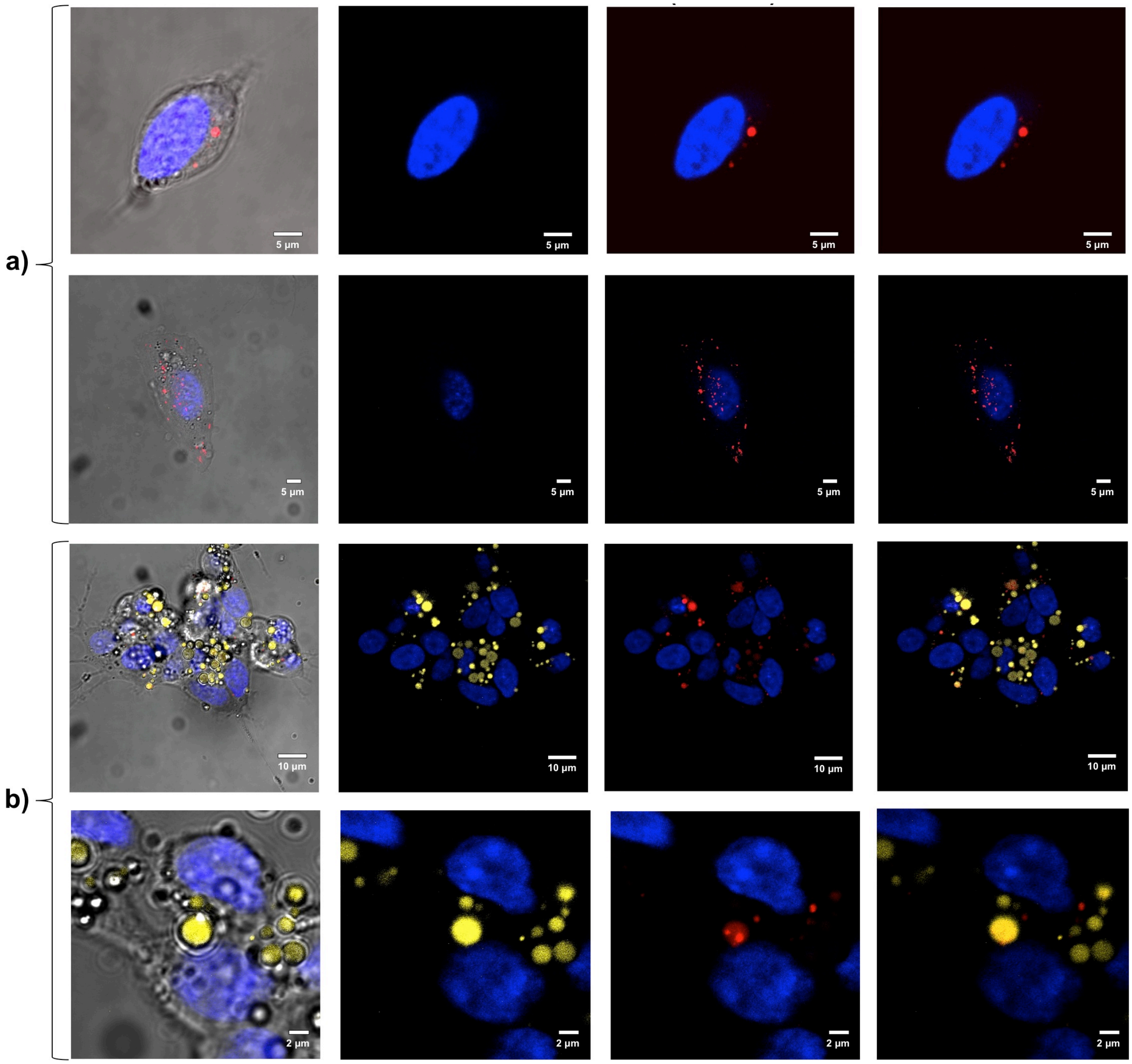

***Figure S13**. Staining with LysoTracker reveal that many **SHS1** colocalize with the endolysosomal vesicles of neuroblastoma (SH-SY5Y) cells. The cells were incubated for 3 days with (a) LysoTracker and (b) LysoTracker + **SHS1** (69.9 µM) with KI (27.9 mM). Columns correspond to: (1) Bright field merged, (2) the yellow channel (561 nm) for the **SHS1** particles, (3) the LysoTracker red channel (633 nm), and (4) fluorescence merged. The emission filters used were BP 575-615 IR (λ_{ex} = 561 nm), BP 420-480 (λ_{ex} = 405 nm for Hoechst showed in all images), and BP 652-738 (λ_{ex} = 633 nm).*

<u>*B5. Macropinocytosis inhibitor (amiloride) studies*</u>

Macropinocytosis inhibition experiments were performed by pre-incubation with incremental amounts of amiloride for 30 minutes, followed by replacement of the cell culture medium with fresh DMEM before the addition of the **SHS1** particles. After incubation for 24 hrs, the cells were washed with PBS, followed by fixation with 4% of formaldehyde in PBS, and addition of mounting media containing DAPI. Subsequent CLSM imaging enabled the determination of the ratio of the **SHS1** particles per cell as shown in the caption of Figure S7.

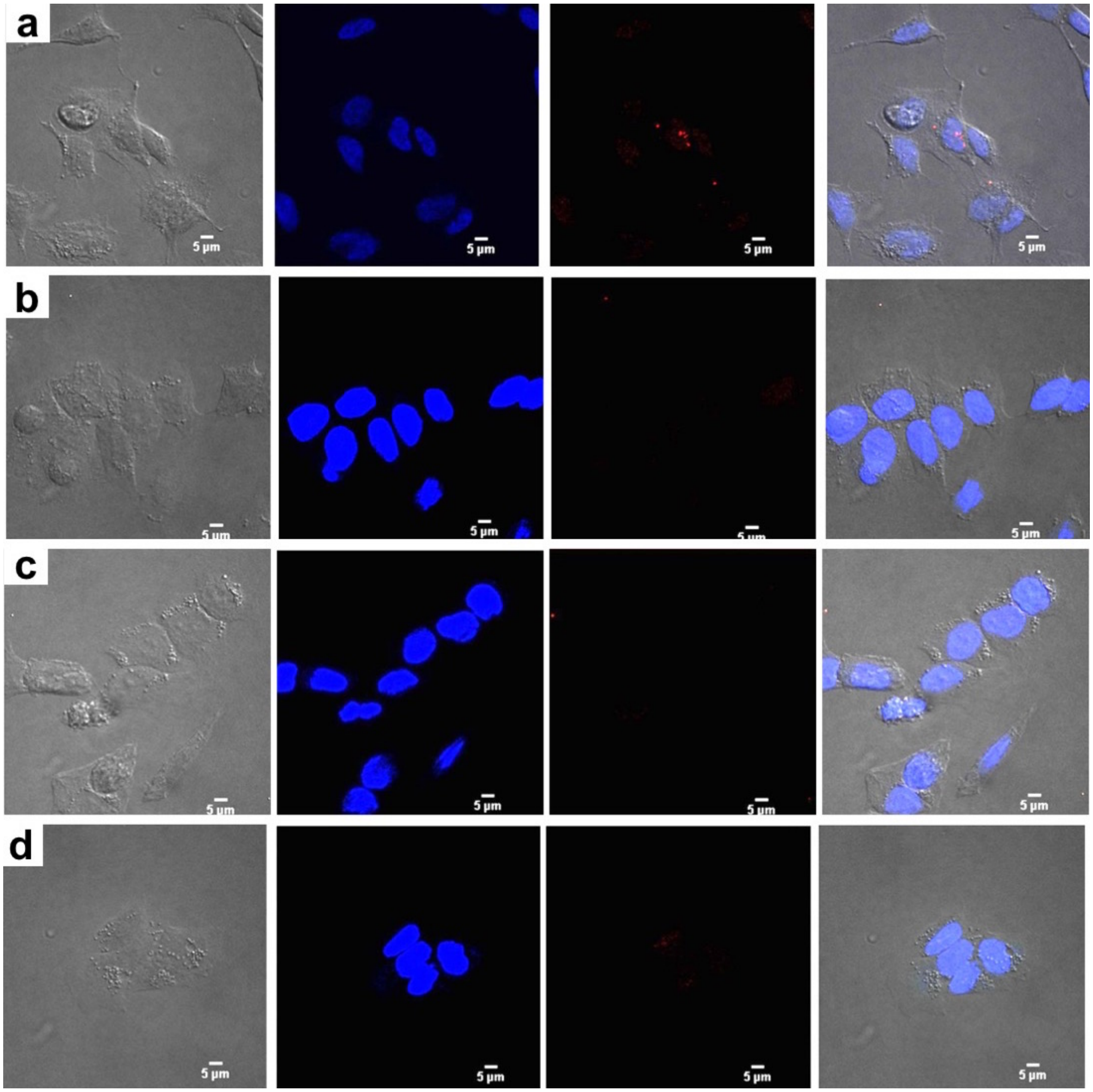

***Figure S14**. CLSM images of SH-SY5Y cells incubated with **SHS1** (200 μL, 303 μM in **1**) for 24 hours. Cells were pre-treated for 30 min with increasing amounts of amiloride: a) 0.0 mM, b) 0.3 mM c) 0.5 mM d) 1 mM. The calculated ratio of **SHS1** particles per cell is (with the percentage relative to (a) in parenthesis): a) 1.95, b) 0.24 (12%), c) 0.25 (13%), d) 0.33 (17%). Columns correspond to: (1) Bright field merged, (2) the blue channel (405 nm) for the Hoechst nuclear stain, (3) the red channel (561 nm) for the **SHS1** particles, and (4) fluorescence merged. The emission filters used were BP 575-615 IR (λ_{ex} = 561 nm) and BP 420-480 (λ_{ex} = 405 nm).*

C. Drug delivery & controlled release (Figs. S15-S20)

C1. In vitro controlled release experiments

Prior to the controlled release experiments, different equivalents of Dox were tested to evaluate the loading capacity and leakage of Dox from **DOX@SHS1**. First, the SHS (150 µL; 5 mM) colloid was formed by increasing the temperature (using a water bath) to 40 °C, which is above the LCST. To each of these solutions, different equivalents of Dox were added as described in the caption of Figure S13. To prepare these samples, different volumes of a solution of Dox (487 µM) were added, which resulted in different final concentrations of SHS, but not its total amount (mmoles) since all samples were prepared using the same initial volume of SHS colloid. After fixing the SHS particles as described in section B, aliquots from the aqueous phase were used to determine the amount of free **DOX** by UV-Vis spectrometry on the **DOX@SHS1** colloid. All the measurements were performed in triplicate for the six solutions of ***DOX@SHS1*** with different Dox equivalents.

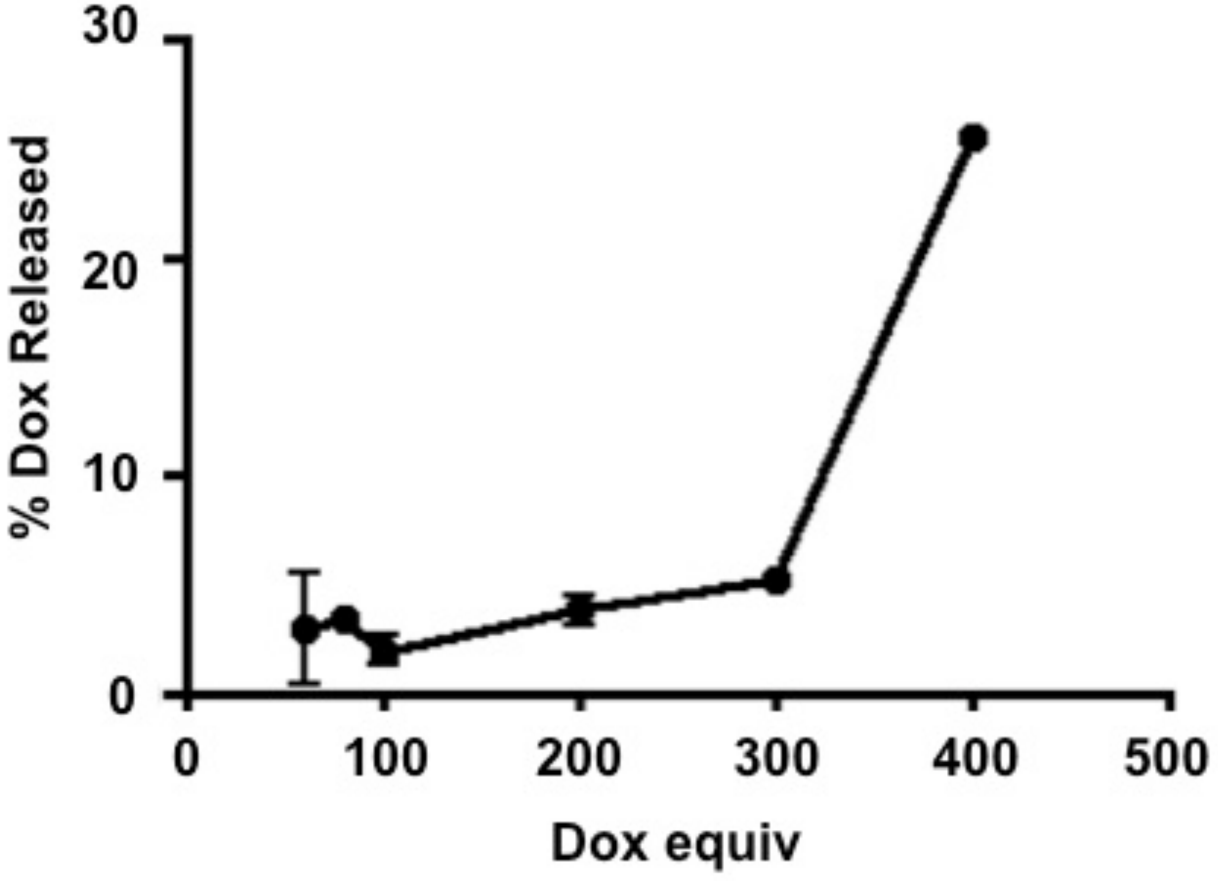

Figure S15. *The loading of **DOX** into **SHS1** affects kinetics of release with a sharp increase at >300 equiv. **DOX** was detected in the aqueous phase at pH 7.2 and 40 °C during 5 min of incubation after the encapsulation of **DOX** (487 µM; 60, 80, 100, 200, 300, 400 equiv) in **SHS1** (0.833 mM) with KI (333 mM). This experiment illustrates the level of leakage of Dox from the **DOX@SHS1** particles. The **DOX** detected in the aqueous phase is assumed to originate from the colloidal phase of **SHS1** particles. The percent of released **DOX** was calculated for each point, by using the concentration of **DOX** detected in the aqueous phase with respect the initial maximum concentration of 487 µM **DOX** added in the colloidal phase of **SHS1**.*

For the time-release experiments, 100 µL of **DOX@SHS1** colloid were added to a 7 mL scintillation vial at 40 °C containing 1.0 mL of PBS (pH 7.2) or sodium acetate buffer (pH 5.0), which resulted in the formation of two phases (colloidal and aqueous non-colloidal). We extracted 100 µL of the aqueous phase at different time points (5 min, 15 min, 30 min, 60 min, 146 min and 24 h) followed by analysis by UV-Vis. These measurements were performed in triplicate for all the six samples at different pH values.

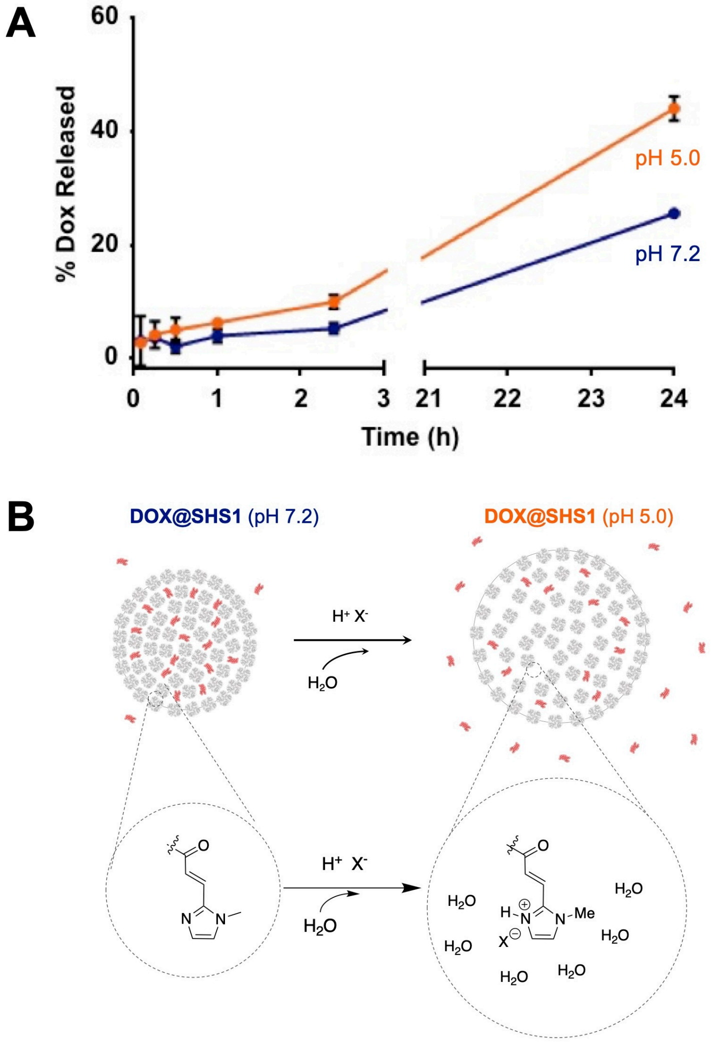

Figure S16. *Enhanced DOX release from **DOX@SHS1** at lower pH. (A) Percentage of doxorubicin released from **SHS1** colloids into the aqueous phase at pH 5.00 ± 0.01 (orange) and pH 7.20 ± 0.01 (blue) after incubation at 40.0 °C. The concentration of **DOX** in the aqueous (PBS) phase was quantified by UV-Vis spectroscopy at 480 nm. All detected **DOX** is assumed to originate from the colloidal phase of **SHS1**. The percentage of released **DOX** was calculated by normalizing the **DOX** concentration in the aqueous phase to its initial concentration in the colloidal formulation (487 µM **DOX**, corresponding to 100 equivalents relative to 0.833 mM **SHS1** in 333 mM KI). (B) Proposed mechanism for pH-dependent release. At lower pH, protonation of the imidazole moieties in the G-derivatives increases the accumulation of counteranions (denoted as* X^-*), generating electrostatic repulsion between the SGQ subunits. This repulsion drives swelling and expansion of the SHS particles, increasing their internal hydration and promoting faster **DOX** release.*

<u>C2. Drug delivery to neuroblastoma cells</u>

C2.1. ***DOX@SHS1*** *sample preparation*

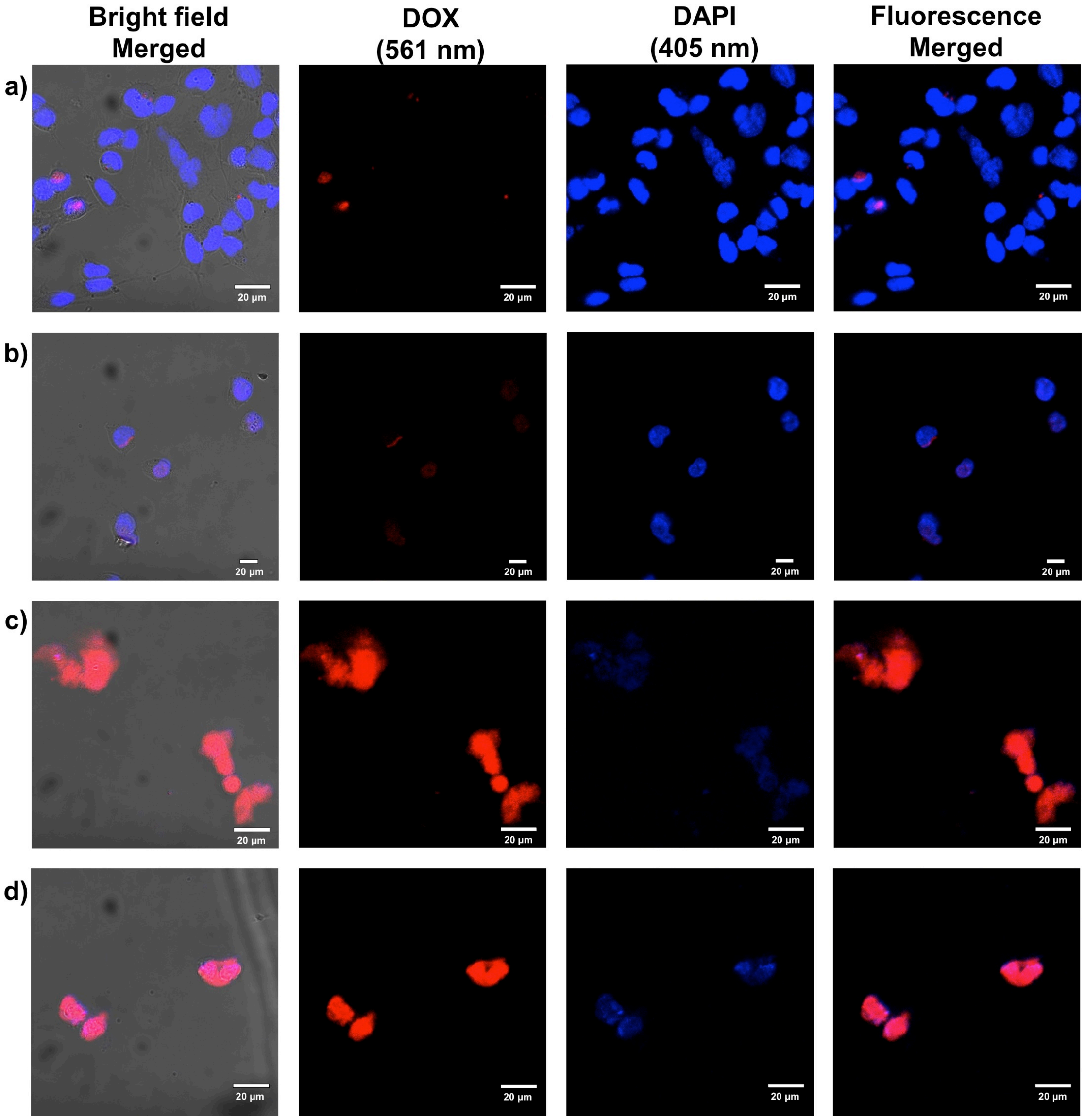

Figure S17. *Uptake of* ***DOX*** *by SH-SY5Y cells after 12 h of incubation. The concentration of* ***DOX*** *for each row was: (a) 0.236 µM; (b) 3.0 µM; (c) 6.5 µM; (d) 40.7 µM. The red channel was used to measure* ***DOX*** *at λ_{ex} = 561 nm (emission filter: 588-738) while the blue channel was used to measure* ***DAPI*** *stain in the cell nuclei at λ_{ex} = 405 nm (emission filter: BP 420-480).*

A 100 µL aliquot of SHS colloid, prepared as described in Section B, was incubated with either 100 equivalents or 8 equivalents of doxorubicin (**DOX**, stock concentration: 487 µM) under stirring at 40 °C for 3 minutes. After dilution into the SHS colloid in PBS, the final **DOX** concentrations were 246 µM (for 100 equiv) and 39 µM (for 8 equiv), respectively. The resulting **DOX@SHS** colloidal suspension was washed with PBS using a sedimentation cone to remove unbound **DOX**, followed by the fixation protocol outlined in Section B. This yielded a final concentration of 303 µM

DOX@SHS1. For cell treatment, 100 µL of the **DOX@SHS1** suspension was added to 1.0 mL of SH-SY5Y cell culture medium and incubated under standard conditions.

For the CLSM images, the Mean Fluorescence Intensity (MFI) for **DOX** delivery experiments was determined by averaging the fluorescence signal of all nuclei in the 561 nm channel. Statistical analyses were performed using GraphPad Prism 10. Data were evaluated via one-way ANOVA with a non-parametric approach, applying Brown-Forsythe and Welch ANOVA tests followed by Dunnett's T3 multiple comparisons test. Group differences were further assessed using the Kruskal–Wallis test, with significance levels reported as follows: $P = 0.5575$ (not significant, pGFP/pCRI transfection), $P > 0.9999$ (not significant, **DOX** delivery), $P = 0.0024$ (**, statistically significant), and $P < 0.0001$ (****, highly significant).

C2.2. Live-cell imaging studies

Live-cell imaging provides provide some insight related to the endocytic mechanism by which the SH-SY5Y cells internalize the **SHS1** and its complexes (e.g., **DOX@SHS1**).

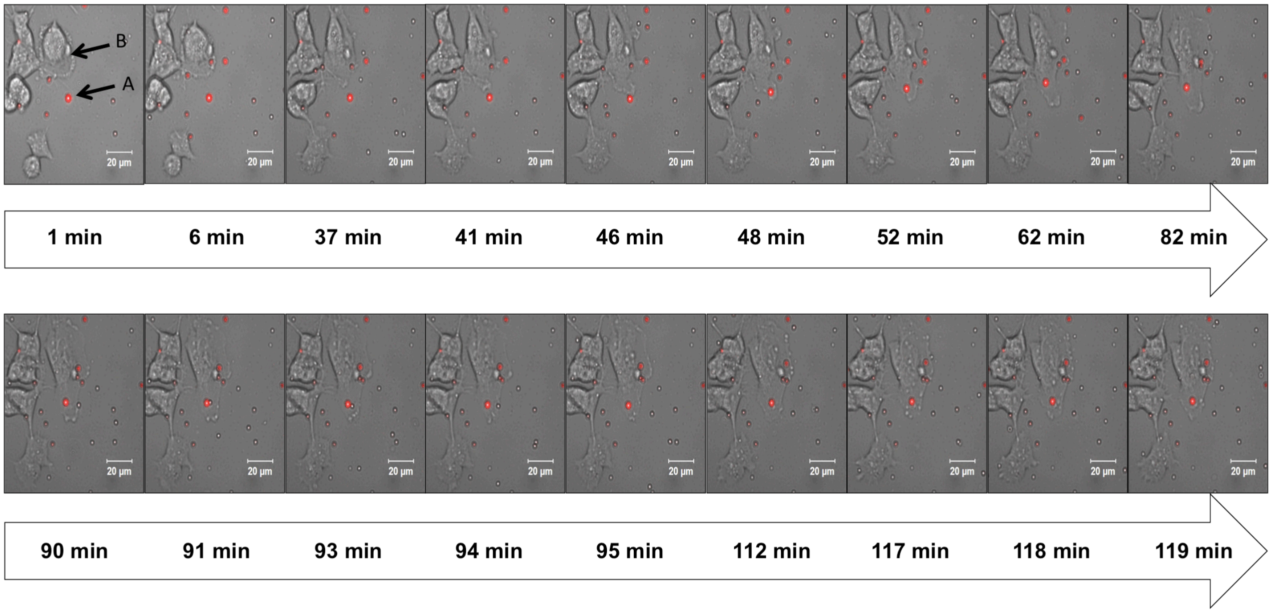

Figure S18. *Live-cell imaging snapshots showing uptake of **DOX@SHS1** by SH-SY5Y cells. In the images, SHS particles are labeled "A" and representative SH-SY5Y cells are labeled "B." SH-SY5Y cells were incubated with **DOX@SHS1** (9.2 µM of **SHS1** preloaded with 100 equiv of **DOX**) in the presence of KI (10.1 mM). The red channel was used to monitor **DOX@SHS1** fluorescence (λ_{ex} = 561 nm; emission filter: LP 575). No nuclear staining was applied (blue channel inactive). See Movie S1 for more details.*

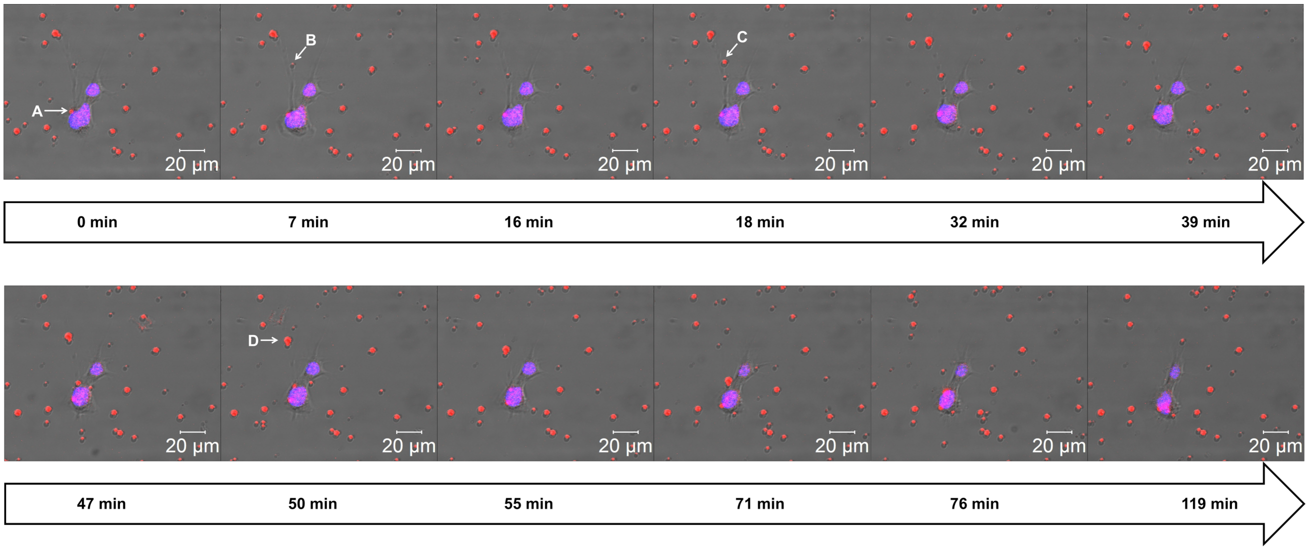

Figure S19. *Live-cell imaging snapshots showing the uptake of* ***DOX@SHS1*** *by SH-SY5Y cells, with stained nuclei for reference. Panels A–D highlight selected* ***DOX@SHS1*** *particles internalized via endocytosis. Cells were incubated with* ***DOX@SHS1*** *(27.5 μM* ***SHS1*** *preloaded with 100 equivalents of* ***DOX****) in the presence of KI (11.0 mM).* ***DOX@SHS1*** *fluorescence was detected in the red channel (λ_{ex} = 561 nm; emission filter: LP 575), while nuclei were visualized in the blue channel using Hoechst stain (λ_{ex} = 405 nm; emission filter: BP 420–480). A Z-stack reconstruction from the end of the experiment is shown in Figure S20. See Movie S2 for more details.*

C2.3. Distribution of ***DOX@SHS1*** *in SH-SY5Y cells by Z-stacking technique*

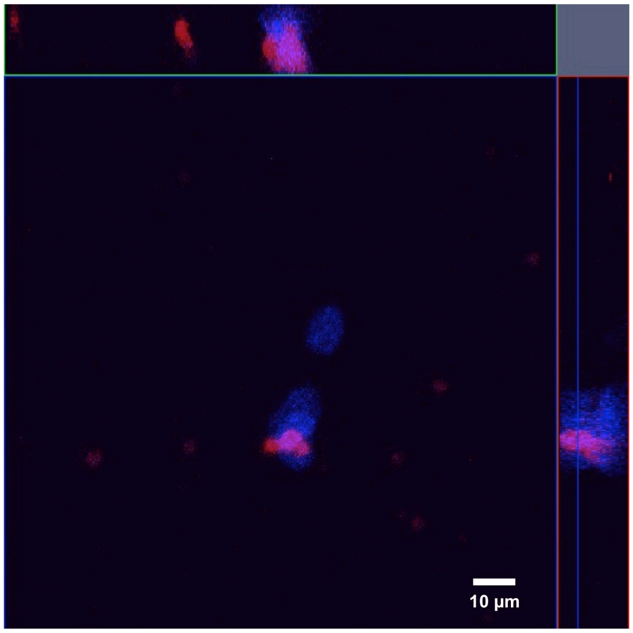

Figure S20. *Z-stack visualization measured at the end of the live-cell imaging experiment shown in Figure S17. This image confirms the intracellular localization of the* ***DOX@SHS1*** *that are in the same confocal plane of the nucleus, which shows Dox colocalization. See the caption of Figure S17 for experimental details.*

D. Gene delivery in vitro (Figs. S21-S25)

D1. General information of plasmid preparation

Transfection experiments were performed in SH-SY5Y neuroblastoma cells using *SHS1* as a gene-delivery agent. The encapsulated plasmids pIRES2-EGFP (**pGFP**) and pE2-Crimson-C1 (**pCri**) were purchased from Clontech Laboratories, Inc. and used without further purification or treatment. A volume of 500 μL of SGQ solution formed as previously described in section B, was incubated with either **pGFP** (150 μL; 574 ng/μL) or **pCri** (50 μL; 480 ng/μL) for 2.5 h stirring at 4–8 °C. The resulting **pDNA@SHS1** complexes were then fixed following the procedure described in section B for a final concentration of 303 μM. Incubation experiments were performed by adding 300 μL of **pDNA@SHS1** to 1.5 mL the cell culture medium.

D2. SH-SY5Y cells incubation with ***SHS1***

The SH-SY5Y cells were seeded 72 hours prior to **pDNA@SHS1** transfection in 2-well cover glass chambers, for a density of 1 x 10^5 cells per well. At the time of the transfection, the medium in each well was replaced with fresh complete medium (1.5 mL of DMEM). The **pDNA@SHS1** and control mixtures were added each into their corresponding wells and incubated with the cells for 3 and 8 days under 5% CO_2 with no movement. Lipofectamine™ 2000 (**LA2K**) was used as a positive control and mixtures were performed by following the manufacturer's instructions.

Hoechst 33342 was used to stain the nuclei of the cells to track where our particle resides inside them. **LA2K** was used as a positive control, while SH-SY5Y neuroblastoma cells alone and in presence of non-encapsulated plasmid as negative controls. For visualization in CLSM we used λ_{ex} = 488 nm laser represented as a yellow channel for **SHS1** (which are fluorescent when excited at this frequency) alone and green for "**SHS1 + pGFP**" for the positive control. For visualization of Crimson in presence of **SHS1**, or for the positive control, we used λ_{ex} = 633 nm in which emission channel, which is represented in red.

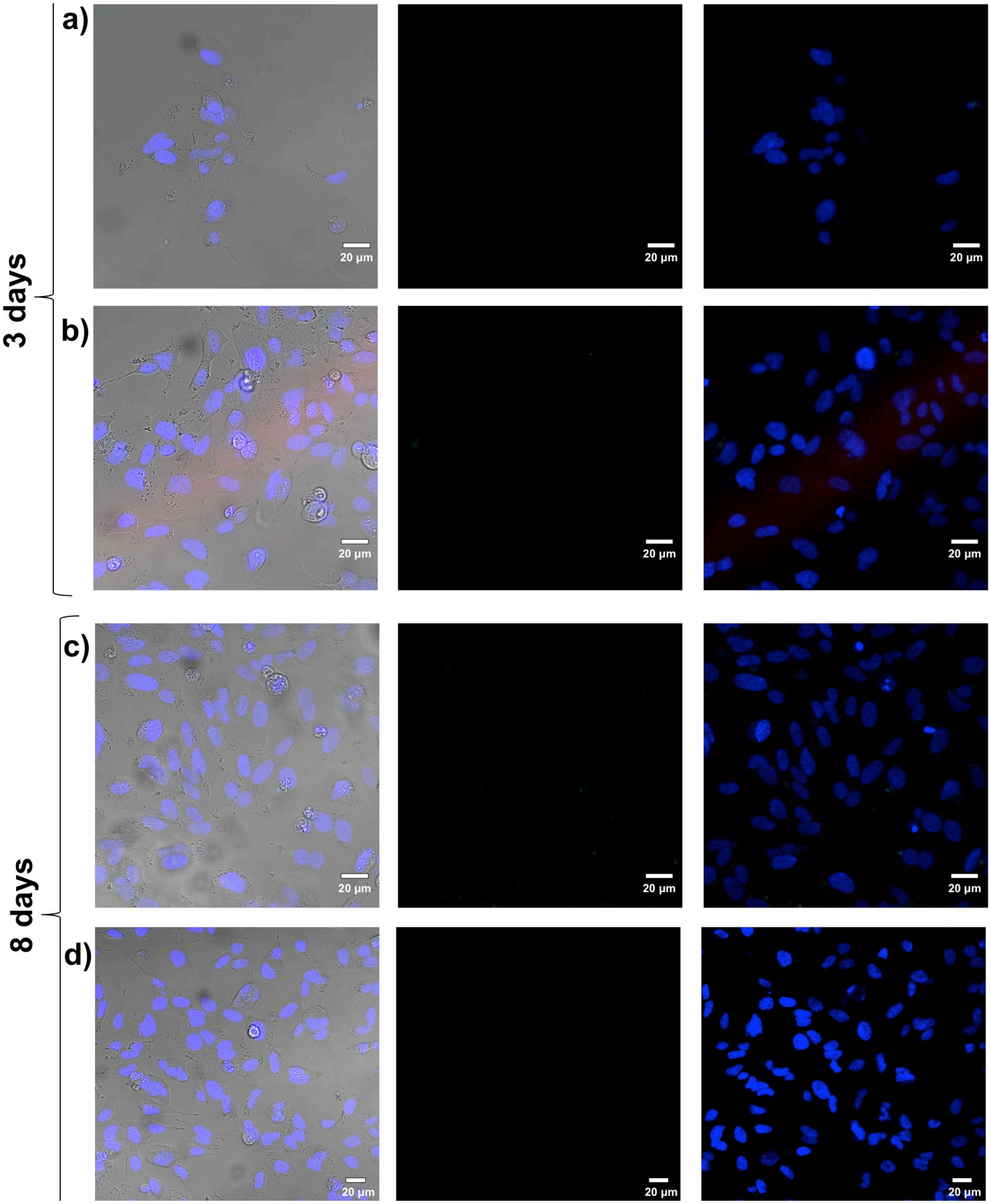

Figure S21. *Negative control images corresponding to transfection experiments of SH-SY5Y cells with (a, c)* ***SHS1*** *particles or (b, d) only* ***pGFP*** *(0.0002 equiv) after 3 days (a, b) and 8 days (c, d). Images were taken after incubation at 37 °C under 5%* CO_2 *without movement. Columns correspond to: (1) Bright field merged, (2)* ***SHS1*** *particles (a, c) or* ***pGFP*** *(b, d), (3) fluorescence merged. Emission filters: Hoechst 33342, BP 420-480 nm;* ***pGFP*** *and* ***SHS1****, BP 505-550 nm.*

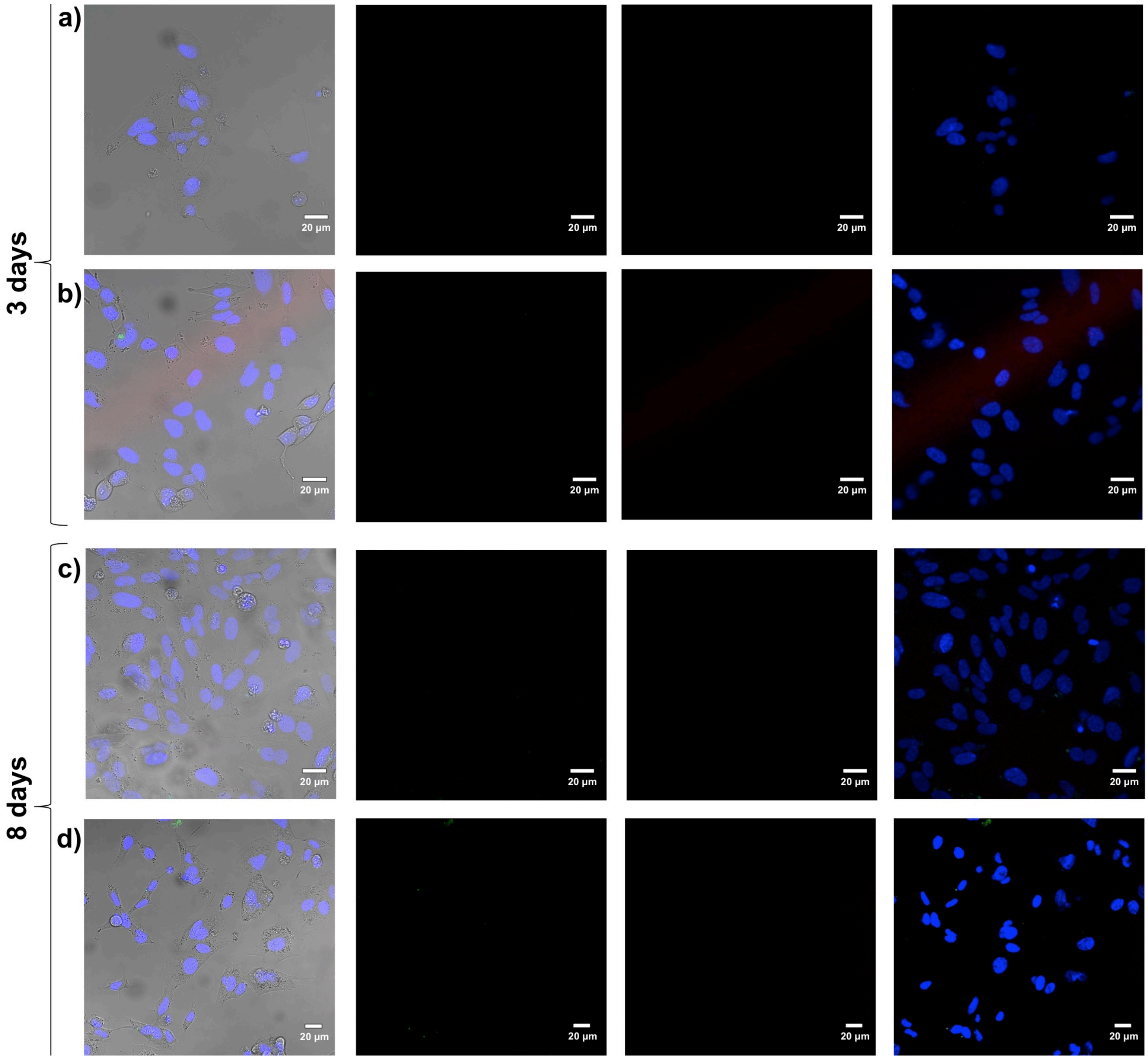

Figure S22. *Negative control images corresponding to the transfection experiments of SH-SY5Y cells with **pCri** (0.0001 equiv) (only b, d) after 3 days (a, b) and 8 days (c, d). Columns correspond to: (1) Bright field merged, (2) the 488 nm channel for the **SHS1** particles, (3) the 633 nm channel for Crimson, and (4) fluorescence merged. Images were taken after incubation at 37 °C under 5% CO_2 without movement. Emission filters: Hoechst 33342, BP 420-480 nm; **SHS1**, BP 505-550 nm; Crimson, BP 652-738 nm.*

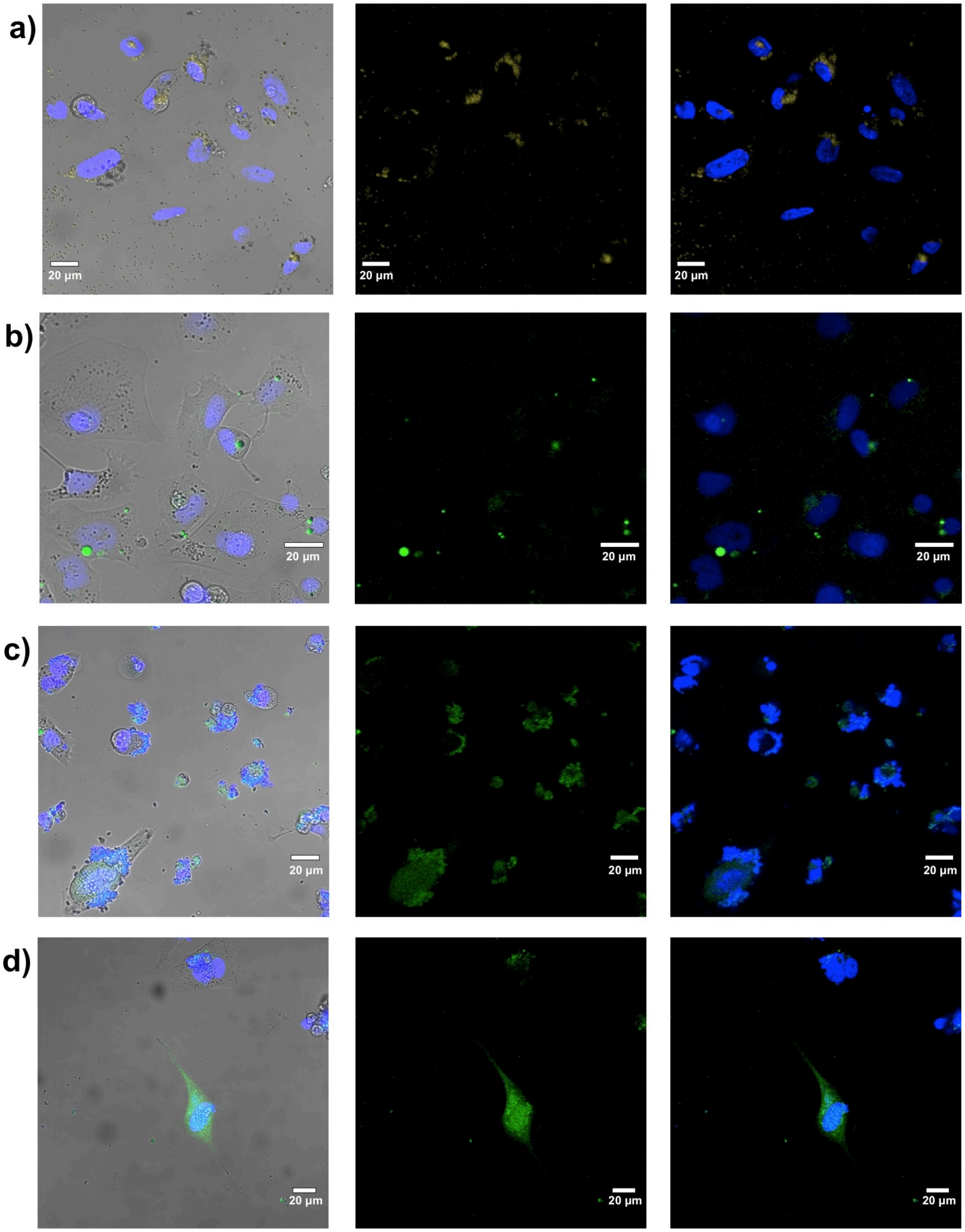

Figure S23. *Transfection of SH-SY5Y cells using* ***pGFP@SHS1****. Conditions: a)* ***SHS1*** *(negative control), b)* ***pGFP@SHS1*** *and c) Lipofectamine with* ***pGFP*** *(positive control) after incubation for 3 days. d) Lipofectamine with* ***pGFP*** *after incubation for 8 days at 37 °C under 5%* CO_2 *without movement (50.5 µM* ***SHS1*** *with 20.2 mM KI, 0.0002 equiv of* ***pGFP****). Columns correspond to: (1) Bright field merged, (2) the 488 nm channel for the* ***SHS1*** *particles and/or for GFP, (3) the blue channel (405 nm) for the Hoechst nuclear stain, and (4) fluorescence merged. The emission filters used were Hoechst 33342, BP 420-480 nm; GFP and* ***SHS1****, BP 505-550 nm.*

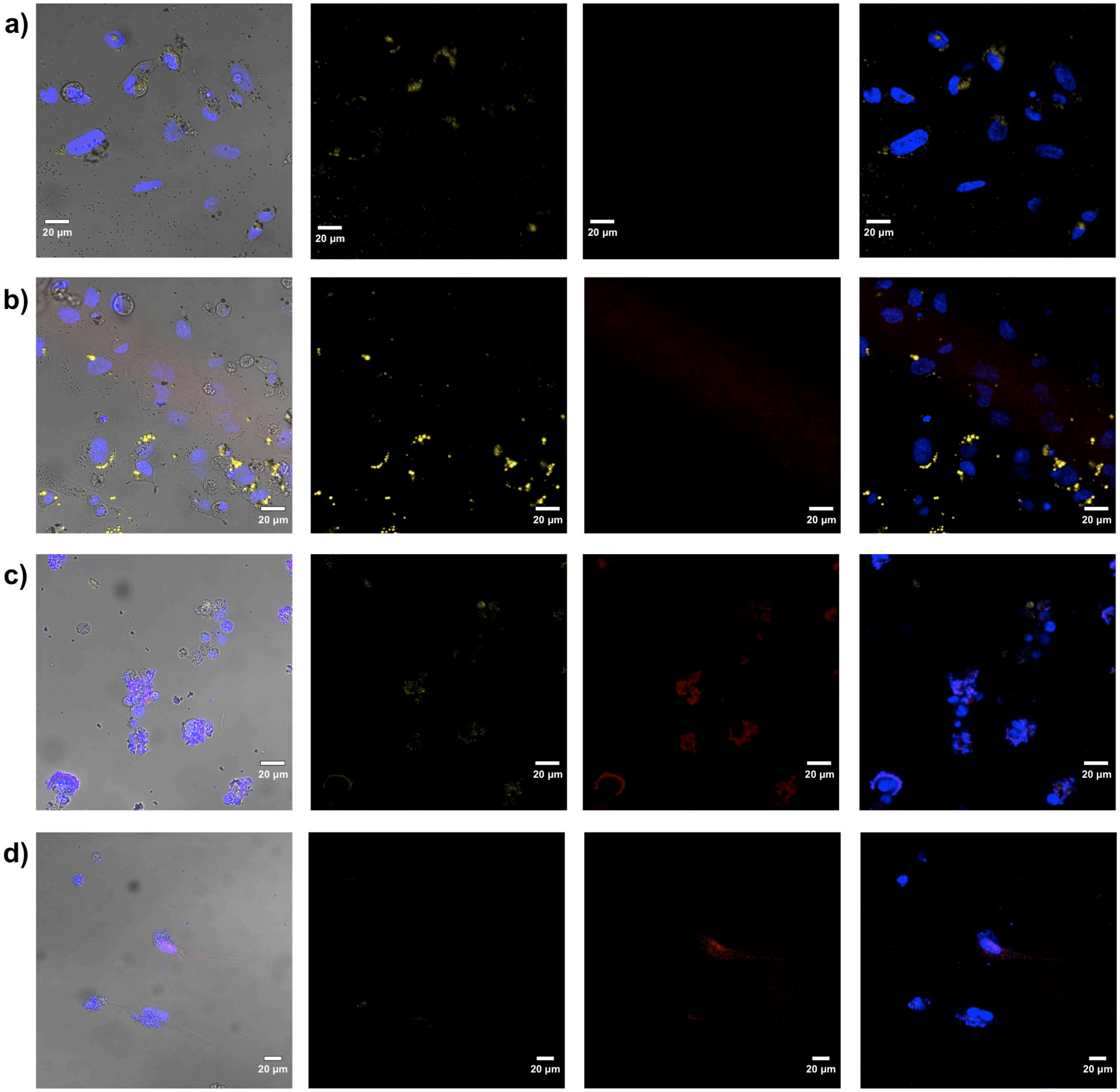

Figure S24. *Transfection of SH-SY5Y cells using* ***pCri@SHS1****. Conditions: a)* ***SHS1*** *(negative control), b)* ***pCri@SHS1*** *and c) Lipofectamine with* ***pCri*** *(positive control) after incubation for 3 days. d) Lipofectamine with* ***pCri*** *after incubation for 8 days at 37 °C under 5%* CO_2 *without movement (50.5 µM* ***SHS1*** *with 20.2 mM KI, 0.0001 equiv of* ***pCri****). Columns correspond to: (1) Bright field merged, (2) the 488 nm channel for the* ***SHS1*** *particles, (3) the 633 nm channel for Crimson, and (4) fluorescence merged. The emission filters used were: Hoechst 33342, BP 420-480 nm;* ***SHS1****, BP 505-550 nm; Crimson, BP 652-738 nm.*

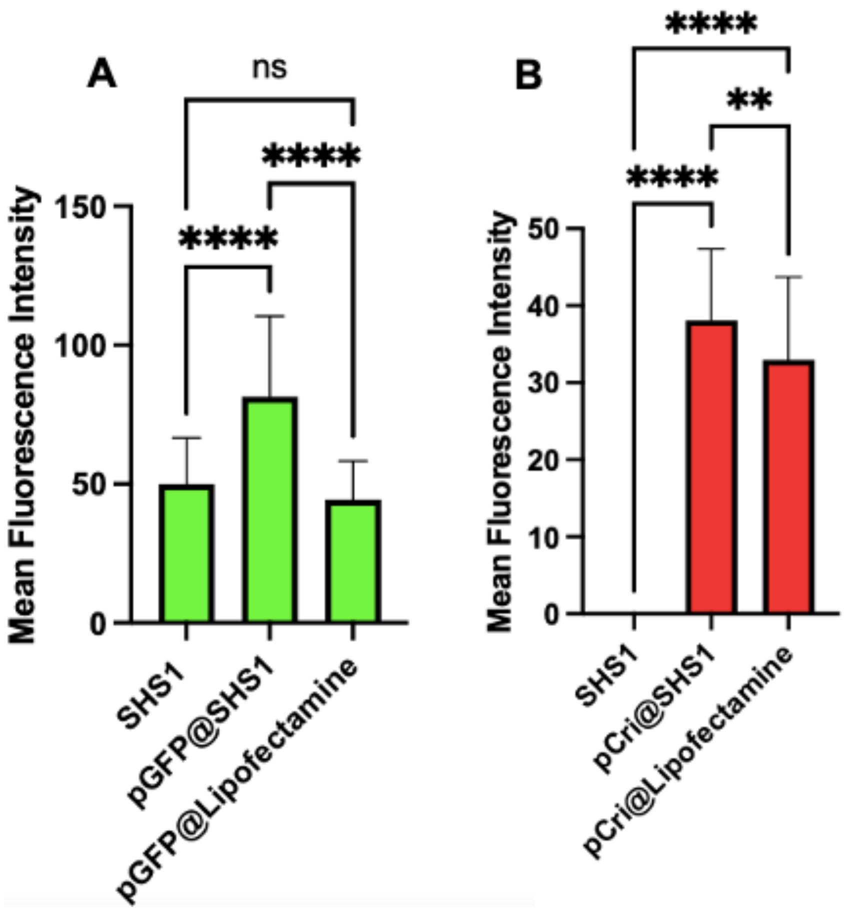

Figure S25. *Mean Fluorescence Intensity (MFI) analysis of transfection experiments in SH-SY5Y cells over an 8-day incubation period. (A) MFI at 488 nm for green fluorescent protein (**pGFP**) and (B) MFI at 633 nm for crimson fluorescent protein (**pCri**), following transfection with either **SHS1**-based complexes or Lipofectamine.*

E. Supporting References

(1) Negrón, L. M.; Díaz, T. L.; Ortiz-Quiles, E. O.; Dieppa-Matos, D.; Madera-Soto, B.; Rivera, J. M. Organic Nanoflowers from a Wide Variety of Molecules Templated by a Hierarchical Supramolecular Scaffold. *Langmuir* **2016**, *32* (10), 2283–2290. https://doi.org/10.1021/acs.langmuir.5b03946.
(2) Prieto-Costas, L. A.; Milton, L.; Quiñones-Jurgensen, C. M.; Rivera, J. M. Screening and Quantification of the Encapsulation of Dyes in Supramolecular Particles. *Langmuir* **2021**, *37* (43), 12681–12689. https://doi.org/10.1021/acs.langmuir.1c02065.
(3) Prieto-Costas, L. A.; Rivera-Cordero, G. R.; Rivera, J. M. Quantifying and Modulating Protein Encapsulation in Guanosine-Based Supramolecular Particles. *Bioconjugate Chemistry* **2023**, *34* (11), 2112–2122. https://doi.org/10.1021/acs.bioconjchem.3c00412.
(4) Betancourt, J. E.; Rivera, J. M. Tuning Thermoresponsive Supramolecular G-Quadruplexes. *Langmuir* **2015**, *31* (7), 2095–2103. https://doi.org/10.1021/la504446k.
(5) Betancourt, J. E.; Subramani, C.; Serrano-Velez, J. L.; Rosa-Molinar, E.; Rotello, V. M.; Rivera, J. M. Drug Encapsulation within Self-Assembled Microglobules Formed by Thermoresponsive Supramolecules. *Chem. Commun.* **2010**, *46* (45), 8537–8539. https://doi.org/10.1039/C0CC04063K.
(6) Negrón, L. M.; Meléndez-Contés, Y.; Rivera, J. M. Patchy Supramolecules as Versatile Tools To Probe Hydrophobicity in Nanoglobular Systems. *J. Am. Chem. Soc.* **2013**, *135* (10), 3815–3817. https://doi.org/10.1021/ja401373h.

*Disclaimer: *The project described was supported by funding from the National Institute of General Medical Sciences. The content is solely the responsibility of the authors and does not necessarily represent the official views of the National Institute of General Medical Sciences or the National Institutes of Health.*
